# Subclinical epileptiform discharges in Alzheimer’s disease are associated with increased hippocampal blood flow

**DOI:** 10.1186/s13195-024-01432-9

**Published:** 2024-04-12

**Authors:** Christian Sandøe Musaeus, Troels Wesenberg Kjaer, Ulrich Lindberg, Mark B. Vestergaard, Henrik Bo, Wiberg Larsson, Daniel Zvi Press, Birgitte Bo Andersen, Peter Høgh, Preben Kidmose, Martin Christian Hemmsen, Mike Lind Rank, Steen Gregers Hasselbalch, Gunhild Waldemar, Kristian Steen Frederiksen

**Affiliations:** 1 Danish Dementia Research Centre (DDRC), Department of Neurology, Copenhagen University Hospital – Rigshospitalet, Inge Lehmanns vej 8, Copenhagen, 2100 Denmark; 2 Regional Dementia Research Centre, Department of Neurology, Zealand University Hospital, Vestermarksvej 11, Roskilde, 4000 Denmark; 3 Functional Imaging Unit, Department of Clinical Physiology and Nuclear Medicine, University of Copenhagen, Valdemar Hansens Vej 13, Glostrup, 2600 Denmark; 4 Department of Clinical Medicine, University of Copenhagen, Blegdamsvej 3B, Copenhagen, 2200 Denmark; 5 Department of Electrical and Computer Engineering, Aarhus University, Finlandsgade 22, Aarhus N, 8200 Denmark; 6 Berenson-Allen Center for Non-invasive Brain Stimulation, Beth Israel Deaconess Medical Center, Harvard Medical School, 330 Brookline Ave, Boston, MA 02215 USA; 7 T&W Engineering, Borupvang 2, Lillerød, 3450 Denmark

**Keywords:** Alzheimer’s disease, Hyperperfusion, Epileptiform discharges, Spike frequency, EEG

## Abstract

**Background:**

In epilepsy, the ictal phase leads to cerebral hyperperfusion while hypoperfusion is present in the interictal phases. Patients with Alzheimer’s disease (AD) have an increased prevalence of epileptiform discharges and a study using intracranial electrodes have shown that these are very frequent in the hippocampus. However, it is not known whether there is an association between hippocampal hyperexcitability and regional cerebral blood flow (rCBF). The objective of the study was to investigate the association between rCBF in hippocampus and epileptiform discharges as measured with ear-EEG in patients with Alzheimer’s disease. Our hypothesis was that increased spike frequency may be associated with increased rCBF in hippocampus.

**Methods:**

A total of 24 patients with AD, and 15 HC were included in the analysis. Using linear regression, we investigated the association between rCBF as measured with arterial spin-labelling MRI (ASL-MRI) in the hippocampus and the number of spikes/sharp waves per 24 h as assessed by ear-EEG.

**Results:**

No significant difference in hippocampal rCBF was found between AD and HC (*p*-value = 0.367). A significant linear association between spike frequency and normalized rCBF in the hippocampus was found for patients with AD (estimate: 0.109, t-value = 4.03, *p*-value < 0.001). Changes in areas that typically show group differences (temporal-parietal cortex) were found in patients with AD, compared to HC.

**Conclusions:**

Increased spike frequency was accompanied by a hemodynamic response of increased blood flow in the hippocampus in patients with AD. This phenomenon has also been shown in patients with epilepsy and supports the hypothesis of hyperexcitability in patients with AD. The lack of a significant difference in hippocampal rCBF may be due to an increased frequency of epileptiform discharges in patients with AD.

**Trial registration:**

The study is registered at clinicaltrials.gov (NCT04436341).

**Supplementary Information:**

The online version contains supplementary material available at 10.1186/s13195-024-01432-9.

## Background

Patients with Alzheimer’s disease (AD) and dementia with Lewy bodies (DLB) are at an increased risk of developing epileptic seizures [1–7]. A common indicator of seizures are epileptiform discharges identified with electroencephalography (EEG). Recently, studies using long-term EEG monitoring have found an increased frequency of epileptiform discharges in patients with AD as compared to healthy controls (HC) [8–10], and those with discharges showed accelerated disease progression [8, 10].

In patients with temporal lobe epilepsy, ictal activity leads to increased regional cerebral blood flow [11–13], while interictal periods are associated with a decreased rCBF [14–16]. Studies in AD have found that epileptiform discharges are more frequent than in HC using long-term EEG monitoring [8–10]. Although epileptiform discharges are not the same as ictal activity, the former are often associated with epilepsy and may also be due to underlying hyperexcitability. As hyperexcitability can be defined as an increased probability of neuronal activation by a certain stimulus [17], it may be less transient compared to ictal activity and may underly the increased number of epileptiform discharges. In general, most studies that measured the rCBF changes in patients with AD have shown a decreased rCBF in the regions affected by neurodegeneration, which includes the hippocampus [18–21]. However, a few studies found increased rCBF in the temporal lobes including the hippocampus [22, 23]. It is unclear whether the conflicting results can be attributed to a biological difference as opposed to methodological differences but if so, it may be due to varying levels of hyperexcitability. Although the epileptiform discharges seen in AD using long-term in-patient EEG monitoring only involve spikes/sharp waves and not ictal activity [8–10], it is possible that a similar hemodynamic response may underly epileptiform discharges if the hyperexcitability is constantly present. This is supported by a study showing hippocampal hyperexcitability [24] but this association has not been investigated.

Ear-EEG is a new method for long-term EEG recording in the out-patient setting. Ear-EEG is a method where EEG signals are recorded from electrodes placed within a customized earpiece inserted into each ear allowing the individual to engage in their daily routine [25–27]. The signals recorded using ear-EEG has been shown to correspond to the temporal electrodes on scalp EEG [25] and a study in patients with temporal lobe epilepsy has shown that epileptiform discharges can be detected with ear-EEG [28]. Recently, the ear-EEG has been shown to be safe and feasible in patients with AD [29] and a recent study using the same cohort as presented here have found a significantly increased spike frequency in AD [30]. Arterial spin labeling MRI (ASL-MRI) is a non-invasive imaging technique that allows for quantitative measurements of rCBF [19, 20, 31–35]. This makes it a useful technique for measuring rCBF in specific brain regions in AD [19, 20, 31–35]. While most studies have shown a decrease in rCBF in temporal lobe structures, a few studies have found an increased rCBF in AD [22, 35]. Although the heterogeneous findings of both increased and decreased rCBF could be due to methodological challenges, it could also be due to a response to an underlying hyperexcitability, which should be explored. By combining these techniques, it is possible to understand the relationship between epileptiform discharges and rCBF in the hippocampus.

The aim of this study was to investigate the association between rCBF in hippocampus and the frequency of epileptiform discharges in patients with AD as measured with ear-EEG. The reason for hippocampus being investigated was due to previous finding of hippocampal hyperexcitability in patients with AD [24]. Our hypothesis was that increased spike frequency as a measure of hyperexcitability was associated with increased rCBF in hippocampus as seen in patients with epilepsy.

## Methods

### Participants

In the current study, patients with mild to moderate AD, and healthy controls (HC) were recruited from the Memory Clinic at Copenhagen University Hospital – Rigshospitalet and the Memory Clinic at Zealand University Hospital, Roskilde. Patients with AD met the NIA-AA criteria for probable AD with amnestic presentation [36] with the diagnosis being determined based on a consensus conference. The consensus conference included information from structural imaging and, in most instances, [^18^F]FDG positron emission tomography (PET). Some patients underwent lumbar puncture (n_AD_=18) with evaluation of amyloid-β42, phosphorylated tau, and total tau while some underwent a [^11^C] Pittsburgh compound-B-PET scan (n_AD_=2) or both (n_AD_=3) as part of the clinical work-up.

In patients with AD, the inclusion criteria included (1) a mini-mental state examination (MMSE) [37] score of 16–28, (2) age between 50 and 90 years, (3) native Danish speaker, (4) at least 7 years of education, (5) hearing and vision sufficient for neuropsychological examination, (6) no alcohol or drug abuse within the last two years, (7) no contraindications for MRI, (8) an MRI or CT scan that supported the diagnosis of AD, (9) the general health conditions of the patient allowed participation in the study (as judged by the principal investigator), and (10) living with a caregiver who was able to assist the patient with the home EEG recordings.

The following exclusion criteria were applied: (1) epilepsy prior to the diagnosis of AD, (2) focal pathology (except AD related atrophy) in the hippocampus, i.e. hippocampal sclerosis, (3) living with a relative with serious illness or impaired activities of daily living since the participant may need help to participate in the study, (4) living in a nursing home, (5) psychiatric (except mild depression) or neurological conditions that affects the brain except AD, (6) currently treated with anti-epileptic medication, tricyclic antidepressants or antipsychotics, (7) daily or almost daily administration of medication with known anticholinergic or adrenergic effect, which may affect cognitive abilities or EEG, (8) large cerebral infarctions or more than four lacunar infarctions on MRI, (9) suffering from facial tics/facial hyperkinetic disorders or (10) daily use of hearing aids.

The HC were recruited from a pool of participants in other studies who had expressed interest in participating in new studies. The inclusion criteria were (1) normal cognition (as judged by the principal investigator), (2) a general health compatible with participation in the study as well as criteria 2–7 as applied in patients with AD. The following exclusion criteria were applied: (1) diagnosed with epilepsy, (2) focal pathology in the brain (except mild hippocampal atrophy) as well as exclusion criteria 5–10 as applied in patients with AD but without AD.

The study was approved by the Capital Region Ethics Committee (H-17,035,751), and by the Danish Medicines Agency (2,017,112,288), and registered at the Data Protection Agency (P-2021-866). All participants gave written and oral informed consent before participating in the study. The study is registered at clinicaltrials.gov (NCT04436341).

### Study design

**Fig. 1:**
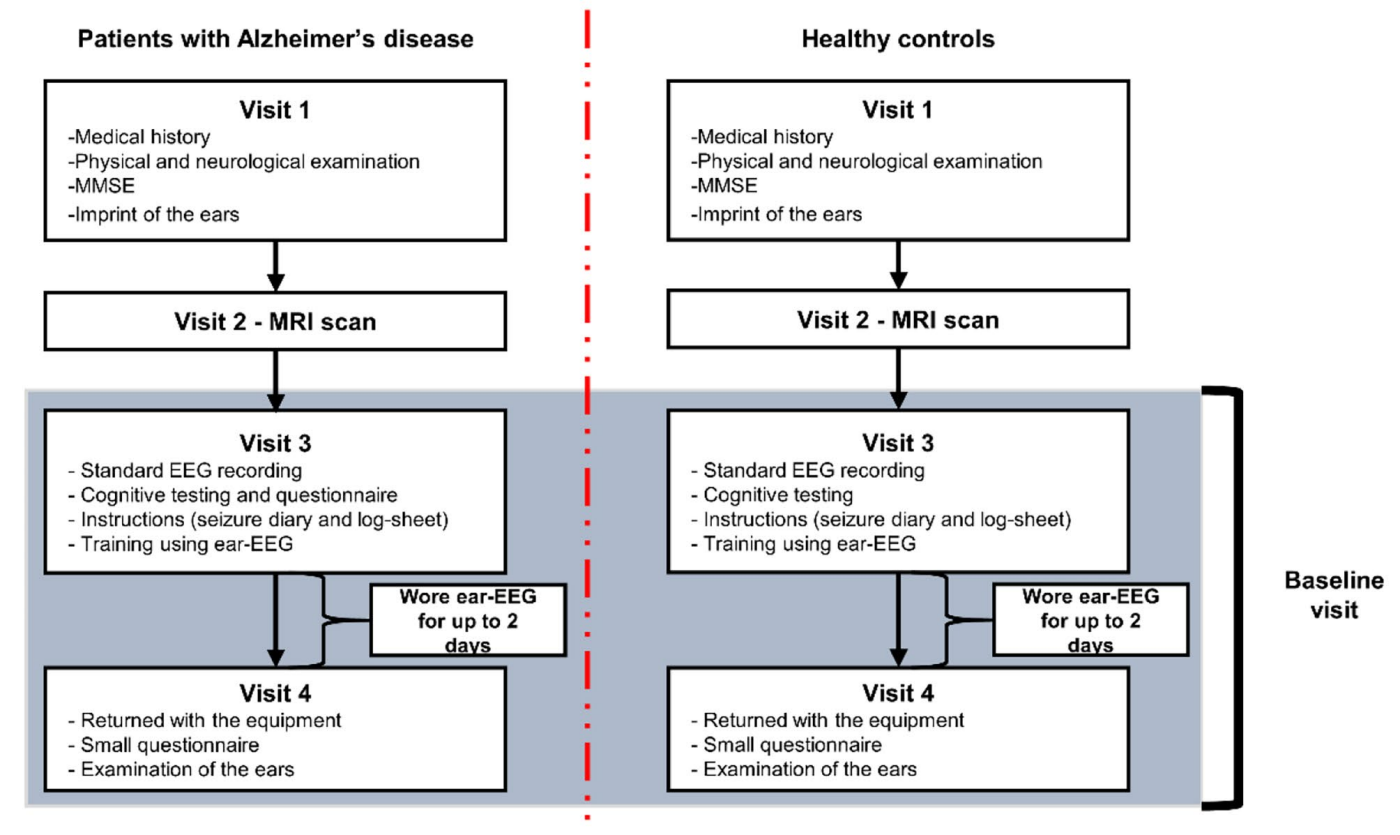
Study design. The patients with AD underwent the same procedures as HC except that no questionnaires were administered for the HC in visit 3

In this cross-sectional study, a total of four visits were planned for patients with AD, and HC, see Fig. 1.

At visit 1, informed consent was obtained followed by assessment of medical history, a physical and neurological examination, the Mini-Mental State Examination (MMSE) (for assessment of global cognitive function) and an imprint of the ears using Otoform A Soft X (Dreve, Germany), a soft ear impression silicone. Subsequently, the patient underwent the following: visit 2) MRI scan (either before or after the ear-EEG recording), visit 3) standard EEG recording together with ear-EEG, Functional Assessment Questionnaire IADL (FAQ IADL) [38] (to assess everyday function), and the neuropsychiatric inventory (NPI) [39] (to assess behavioral and psychological symptoms).

### Ear-EEG recording and review

The participants underwent up to two days of out-patient ear-EEG recording. A full description of the ear-EEG equipment and pre-processing can be found in the supplementary material.

A sharp asymmetric negative potential of 20–200 ms duration was considered an epileptiform discharge (spike/sharp wave) if it was clearly distinct from ongoing background activity and unlikely to be artifactual [40]. Due to the nature of ear-EEG, we could not investigate the spatial distribution of the spikes/sharp wave. All annotations performed by CSM underwent review by a board-certified clinical neurophysiologist (TWK), who made the final ruling. The term epileptiform discharges cover both spikes and sharp waves and assumes an underlying irritative process as seen in epilepsy, even if this cannot be stated with absolute certainty. Both CSM and TWK were blinded to the diagnosis when reviewing the EEGs.

The spike frequency was calculated by dividing the number of spikes by the amount of time (in days) when data from at least one electrode in each ear was being recorded.

The results from the ear-EEG recordings from the patients with AD and HC have been presented elsewhere [30].

### MRI acquisition

All scans were recorded on a 3T Achieva dStream (Philips, Best, The Netherlands) with a 32-channel head receive coil.

### Structural image acquisition and analysis

A sagittal 3D T1-weighted magnetization prepared rapid acquisition gradient echo (T1-MPRAGE) was recorded with the following acquisition parameters: repetition time (TR) 6.9 ms, echo time (TE) 2.82 ms, flip angle 9, matrix size 256 × 255, 155 slices, voxel size 1.1 × 1.1 × 1.1 mm^3^, The T1-weighted images were segmented using Freesurfer (version 7.2.0, https://surfer.nmr.mgh.harvard.edu/) and the volumes for each hippocampus and the inferior lateral ventricles were obtained. Both were normalized to the intracranial volume (ICV). The estimated total intracranial volume (eTIV) generated by FreeSurfer was used as an estimate for ICV in this study as has previously been shown [41].

### Phase contrast mapping

The mean global cerebral blood flow (CBF) was obtained using velocity sensitive phase contrast mapping (PCM) MRI [42, 43]. Blood velocity contrast maps were acquired by a turbo field echo sequence. Measurements were acquired from an imaging plane perpendicular to the carotid arteries and one perpendicular to the basilar artery.

The blood flow in both internal carotids and the basilar artery was calculated by multiplying the mean blood velocity by the cross-sectional area from regions of interest defining each vessel. The global mean CBF was calculated by normalizing the total blood flow from each artery to the total brain weight, which was estimated from the segmentation of the structural MRI images with an assumed brain density of 1.05 g/mL. Calculations were performed using a custom-built script in Python (https://github.com/MarkVestergaard/PCMCalculator).

### Arterial spin labeling MRI analysis

A pseudo continuous ASL (PCASL) sequence with Look-Locker Echo Planar Imaging was chosen. The labeling plane was placed across the neck 9 cm beneath the center of the imaging slab and the labeling duration was 1650 ms. The acquisition parameters were: 13 slices, TE 10.8 ms, voxel size 3.44 × 3.44 × 6.6 mm^3^, FoV 220 × 220 × 85 mm^3^, TR was 300 ms, Look-Locker Flip-Angle 40^o^, slice acquisition duration 22 ms, SENSE factor 2.3, The post-labeling delays were set at [100, 400, 700, 1000, 1300, 1600, 1900 ms]. Each ASL pair (label and control) took 8 s. In the current study, the region of interest was the hippocampus, which resulted in the acquisition plane being placed parallel to the inferior lateral ventricles. After each ASL scan, a single equilibrium magnetization scan (M0) was acquired with the same parameters as the previously described ASL images except for a 10,000 ms TR.

ASL images were quantified using BASIL in FSL (FMRIB software library, version 6.0.5.1, www.fmrib.ox.ac.uk) with quantification [44] and fitting of the macrovascular compartment [45]. The initial prior of bolus arrival time was adjusted to account for the delayed arrival as seen in our sample, which resulted in a selected arrival time prior of 1.6 s. Lastly, the ASL data were registered to the T1wscan. The ASL quantification was not corrected for differences in hematocrit values.

To quantify rCBF in the hippocampus, we first extracted all the values within the hippocampus ROI segmented with FreeSurfer and then removed the voxels with zero values as these were assumed to be contaminated by CSF. Afterwards, any values more than two standard deviations above the mean were removed since they were assumed most likely to represent arteries. Finally, the median value of CBF was extracted for each hippocampus and normalized to the global mean CBF as measured with PCM and the mean of the two values were computed. Due to the both the structural and functional connectivity between the hippocampus and the precuneus [46], we wanted to investigate if a similar association between spike frequency and rCBF in precuneus was present. Here, the same approach for computing the normalized rCBF in the precuneus was used. No other regions were investigated.

### Statistics

The statistical analyses were performed in RStudio (v1.2.1335). When comparing age, education, MMSE, and the time difference between the ear-EEG recording and MRI scan, we performed t-test between AD and HC. Chi-squared tests was performed for testing for sexual distribution.

When comparing the hippocampus volume, global cerebral blood flow, and rCBF in the hippocampus or precuneus, we performed a t-test between AD and HC. Here, the distribution of the data as well as variance between groups were investigated before performing t-tests.

When comparing the number of spikes or sharp waves/24 hours (spike frequency) between HC, and AD, we calculated the rate ratio using the function *rateratio* from the *epitools* toolbox.

Simple linear regression was used to test if spike frequency significantly predicted the normalized mean rCBF in the hippocampus in patients with AD. Since it has been hypothesized that the number of epileptiform discharges increases with AD severity, we performed the same analysis with MMSE as a covariate. In the exploratory analysis, we tested if the spike frequency was associated with the normalized rCBF in the precuneus and whether a similar association between rCBF and spike frequency could be seen in HC.

The R code and output from the subsequent analyses can be found in the supplementary material.

When conducting the voxel-to-voxel analysis to compare normalized rCBF between HC and AD, we performed two-sample unpaired t-tests in randomize from FSL with cluster correction.

## Results

### Patient characteristics

A total of 25 patients with AD, and 15 HC were included. One patient with AD had less than one hour of ear-EEG data after pre-processing and was not included in the linear regression analyses. The average time from MRI scan to ear-EEG recording was 19.18 days and was not significantly different between groups (*p*-value = 0.332). Both left and right hippocampus were significantly smaller in patients with AD as compared to HC (*p*-value < 0.05). See Table 1 for baseline demographics.

**Table 1:** Baseline demographics

	Healthy controls	Alzheimer’s disease	*p*-value
Number of participants	15	25	
Age, mean (SD)	69.5 (7.93)	70.3 (7.79)	0.723
Males/females	8/7	15/10	0.680
Education, mean (SD)	15.2 (2.04)	14.3 (3.08)	0.219
Cholinesterase inhibitor, n (%)	0	24 (96%)	
SSRI/SNRI, n (%)	1 (7%)	4 (16%)	
MMSE, mean (SD)	29.3 (0.88)	23.4 (3.29)	< 0.001
NPI		4.83 (4.06)	
FAQ IADL		14.28 (5.78)	
Time in days between MRI and ear-EEG, mean (SD)*	21.4 (12.46)	17.8 (10.26)	0.332
Left hippocampus volume, % of ICV, mean (SD)	0.26 (0.03)	0.21 (0.03)	< 0.001
Right hippocampus volume, % of ICV, mean (SD)	0.26 (0.3)	0.21 (0.03)	< 0.001

SSRI/SNRI: Selective Serotonin Reuptake Inhibitor/ Serotonin and Noradrenaline Reuptake Inhibitor, MMSE: Mini-Mental State Examination, ADL: Activities of Daily Living, NPI: Neuropsychiatric Inventory, Time in days refers to the number of days between first day of ear-EEG recording and MRI scan in patients who were included in the regression analysis, SD: Standard deviation, ICV: intracranial volume, * Only for the 24 patients with AD with sufficient ear-EEG data

### Spike frequency measured with ear-EEG

The spike frequency was significantly higher in patients with AD (range: 0–13.04 (mean: 3.03 spikes/24 hours) as compared to HC (range: 0–6.66 (mean: 1.04 spikes/24 hours) with a risk ratio of 2.9 (CI: 1.77–5.01, *p*-value = < 0.001).

### Global and regional CBF

Global cerebral blood flow was not significantly (*p*-value = 0.872, t-value = 0.16, df = 38) different between AD (mean (SD): 43.23 ml/100 g/min (12.28)) and HC (mean (SD): 42.60 ml/100 g/min (11.01)).

**Fig. 2:**
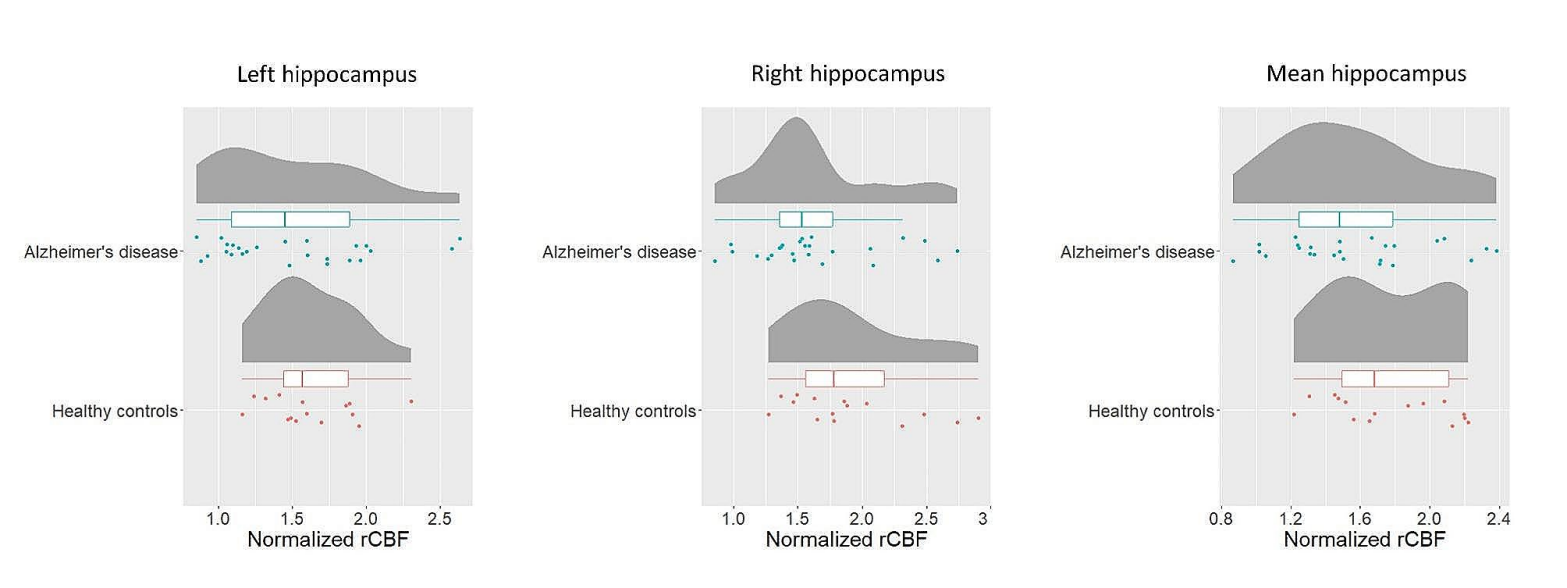
Raincloud plots showing mean normalized rCBF. No significant differences were found between AD, and HC for left hippocampus, right hippocampus, or mean hippocampus

No significant differences were found for the normalized rCBF value in the hippocampus between the AD, and HC for left hippocampus (*p*-value = 0.367, t-value = -0.91, df = 38), right hippocampus (*p*-value = 0.092, t-value = -1.73, df = 38), or mean hippocampus (*p*-value = 0.118, t-value = -1.60, df = 38). See Fig. 2. A significant difference was found between AD and HC for the normalized rCBF in the precuneus (*p*-value = 0.010, t-value = -2.73, df = 38) with a higher normalized rCBF in the HC, see Supplementary Fig. 1.

**Fig. 3:**
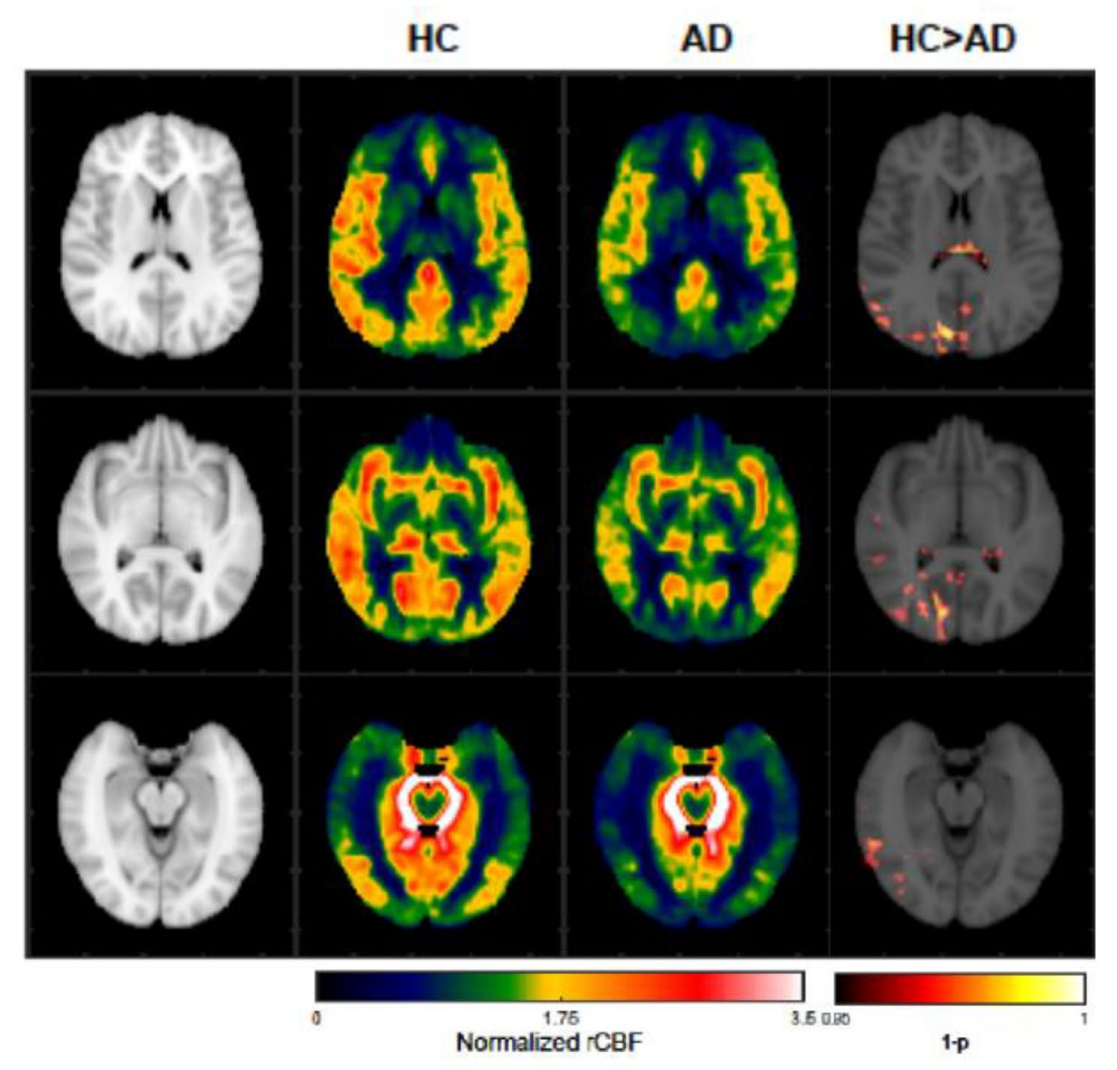
Exploratory analysis of rCBF. A significantly decreased normalized rCBF in the temporal and parietal lobes (including precuneus) in AD as compared to HC

In an exploratory manner, we conducted a voxel-to-voxel comparison and found significant bilateral decreased rCBF in the temporal and parietal lobe in patients with AD as compared to HC, see Fig. 3.

### Association between ASL and spike frequency

**Fig. 4:**
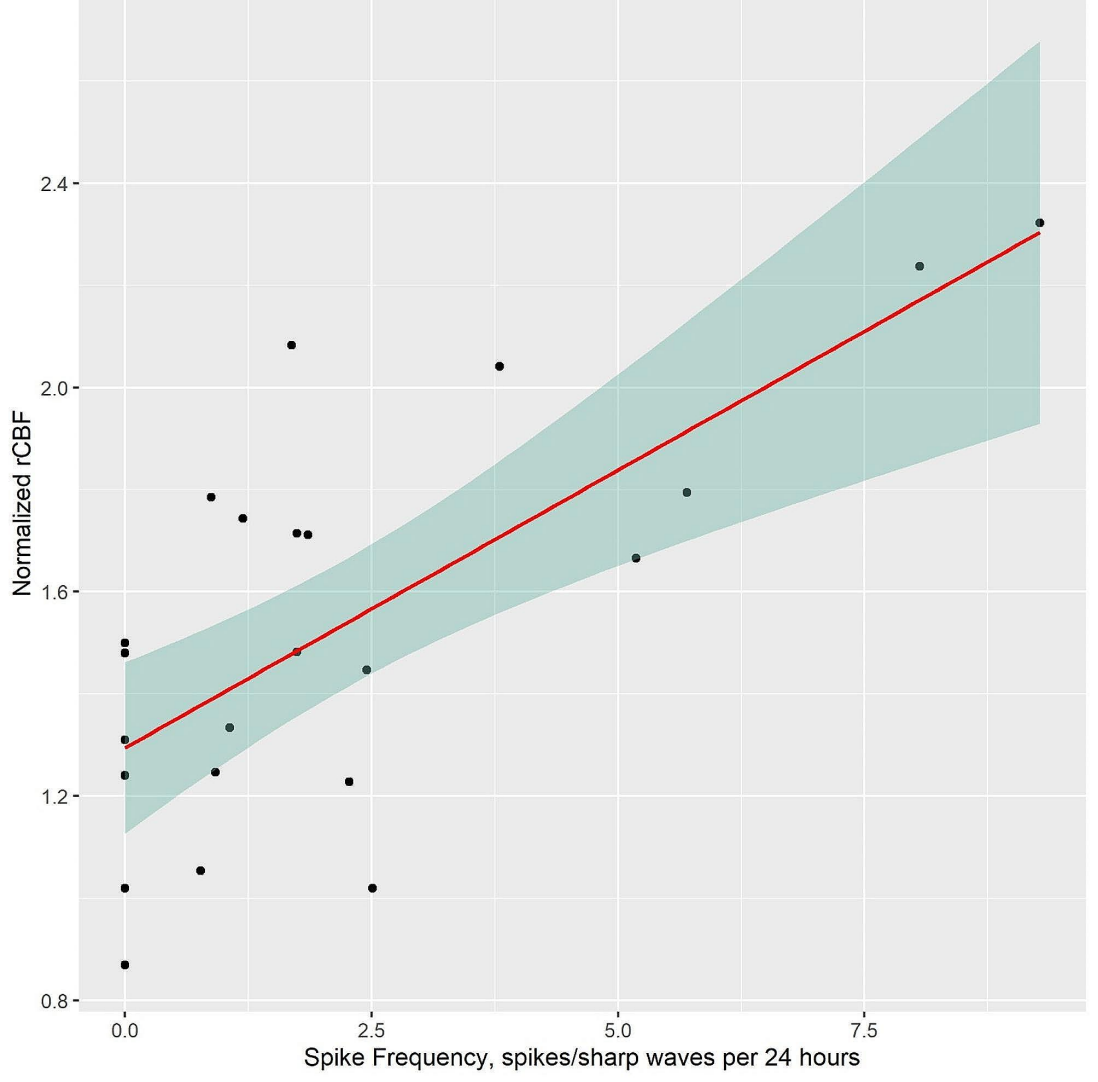
Association between normalized rCBF in hippocampus and spike frequency as measured with ear-EEG. Simple linear regression was applied to test if spike frequency was associated with the normalized mean rCBF in the hippocampus in patients with AD (*p* < 0.001)

A significant positive linear association (estimate: 0.109, t-value = 4.03, *p*-value < 0.001) between spike frequency and normalized rCBF in the hippocampus was found for AD, see Fig. 4. This effect was also present when the outlier was kept in the model (*p*-value = 0.028). See Supplementary Fig. 2 and page 4–7 in the supplementary material for detection of outliers. In the following analysis with MMSE as a covariate the association between spike frequency and rCBF was significant (*p*-value < 0.001) but MMSE was not significant (estimate = 0.006, *p*-value = 0.756), indicating that its inclusion did not significantly alter the association between spike frequency and rCBF. No significant association between rCBF and spike frequency was found for the HC (estimate: 0.021, t-value = 0.491, *p*-value = 0.632), see Supplementary Fig. 5.

As part of the exploratory analysis, we also found a positive association between normalized rCBF in precuneus and spike frequency in patients with AD (estimate: 0.069, *p*-value = 0.037), see Supplementary Fig. 6. This may be explained by the positive association between normalized rCBF in precuneus and hippocampus (estimate: 0.504, *p*-value = 0.013), see Supplementary Fig. 7.

## Discussion

In the present study, we investigated the association between rCBF in the hippocampus and epileptiform discharges (in the form of spike/wave activity) in patients with AD. As hypothesized, a significant linear association between spike frequency and the normalized rCBF in the hippocampus was found in patients with AD. This association was not affected by adjusting for disease severity as measured with the MMSE. In an exploratory analysis a significant positive association between spike frequency and the normalized rCBF in precuneus was found in patients with AD. Spike frequency was significantly higher in patients with AD compared to HC. No difference in the mean hippocampal rCBF was found between AD and HC. The exploratory analysis revealed a significantly lower normalized rCBF in the parietal lobes including precuneus in AD as compared to HC.

The findings of an association between spike frequency and normalized rCBF in the hippocampus suggests that these two biological phenomena may be coupled in AD. Similarly, in patients with epilepsy, rCBF increases at the epileptic focus during the ictal phase [11–13]. Even though we only measured spikes/sharp waves and not ictal activity, the results indicate a common hemodynamic response to epileptiform discharges in both conditions. Due to this resemblance with epilepsy, it is likely that the increasing rCBF in hippocampus with more epileptiform discharges is linked to an increased metabolic demand [47], which may again be linked to accelerated neurodegeneration although this remains speculative. The epileptiform discharges measured in patients with AD in this study may represent a hyperexcitable state of neurons in the hippocampus that does not directly resemble the pathophysiological mechanisms in epilepsy. Using a more sensitive measuring technique, which enables recordings of much smaller amplitudes of spikes, it was found that these discharges are much more common in the hippocampus as compared to what is detected using scalp EEG and possibly indicating a hyperexcitable state [24]. Such a hyperexcitable state may also represent a less transient state than seen during an epileptic seizure. This would also explain the positive association despite the difference in time between ASL acquisition and ear-EEG recording. Furthermore, the association between hippocampal rCBF and spike frequency in HC was not significant, which suggests that this coupling is specific to AD, but more studies are needed. To test the effect of anti-seizure medication on this hyperexcitability, a recent study investigated the changes in rCBF in patients with AD after administration of levetiracetam using ASL-MRI but without long-term EEG monitoring and found an increased hippocampal rCBF [48], which is in contrast to the current findings. Based on the current findings of an association between spike frequency and hippocampal rCBF, we would expect a decrease in rCBF after administration of levetiracetam due to a decrease in the hippocampal excitability. Due to the limited studies in this field, further investigations of the rCBF response to anti-seizure medications while performing long-term EEG monitoring are needed to better understand the relationship between epileptiform discharges and rCBF in AD. Specifically, it remains to be determined how chronic administration of anti-seizure medication in patients with AD will alter hippocampal perfusion and if increased perfusion due to epileptiform discharges will be decreased (i.e., normalization) after administration of anti-seizure medication in patients with AD.

In the current study, no significant difference in rCBF was observed between AD and HC. In the existing literature investigating changes in rCBF in AD using ASL [19, 20, 22, 31–35] for the most part found decreased rCBF have been reported while a few studies found increased rCBF in temporal lobe structures as well as hippocampus [22, 35]. Hippocampal rCBF is affected by both technical (e.g., partial volume effect due to atrophy) and physiological factors that may lead to the heterogeneous findings in the literature. As for the physiological role, the rCBF in AD could be driven by epileptiform discharges leading to a pathophysiological increased rCBF and a lack of difference between the two groups.

In the exploratory analysis, we found significantly decreased normalized rCBF in the precuneus in patients with AD as compared to HC. This could be due to the neuropathological abnormalities being present in the precuneus in the early stages of the disease [49]. Using resting-state functional MRI, the precuneus is considered a main hub of the default mode network and is both structurally and functionally connected to the hippocampus [46, 50] and lower default mode network connectivity has been linked to faster cortical thinning in older adults with amyloid depositions [51]. Due to the interconnectivity between the precuneus and the hippocampus, we investigated the role of the precuneus in epileptiform discharges and found a positive association between spike frequency and rCBF in the precuneus in patients with AD. Another explanation for the association is that the precuneus has reduced rCBF from the deposition of amyloid, as the precuneus is one of the earliest regions to show amyloid deposition in AD, but that the epileptiform discharges lead to a relative increase from a lower baseline. It is possible that the epileptiform discharges measured with ear-EEG in the study originate from the precuneus, but as previous studies have found that most epileptiform discharges originate from the temporal lobes and due to low spatial resolution of ear-EEG, it is most obvious to conclude that the epileptiform discharges originate from the temporal lobes. However, more studies are needed to understand the role of the intrinsic network between hippocampus and precuneus in relation to epileptiform discharges.

The study has several limitations. Due to the focus on the hippocampus, we did not acquire ASL images from the whole brain, which limits further exploration into the possible association between epileptiform discharges and other brain regions. In addition, the MRI acquisition was not performed on the same day as the beginning of the ear-EEG recording, which may lead to the association being weaker due to variability in the measured epileptiform discharges. Although we do not have any knowledge on the day-to-day variations of hippocampal rCBF in patients with AD, it is possible that it may have affected the results. To overcome this, future studies should arrange the study visits to limit any time lag when comparing the two modalities to better understand the association. Furthermore, due to the low spatial resolution of the ear-EEG, it will be of importance to systematically compare epileptiform discharges between ear-EEG and scalp EEG, which was not possible in the current study since only 30 min of scalp EEG was recorded. Although scalp EEG is the gold standard for detection of epileptiform discharges, a study has shown that scalp EEG is unable to detect a large proportion of the epileptiform discharges [24], which may be an advantage of ear-EEG but more studies are needed. Although ear-EEG has been able to detect epileptiform discharges in patients with epilepsy [28], no studies have so far directly compared ear-EEG to scalp EEG in detection of epileptiform discharges. Therefore, it is possible that accuracy is lower with ear-EEG than conventional scalp EEG. Lastly, we did not perform any measurement of AD biomarkers or more extensive neuropsychological examination of the HC and therefore subtle cognitive decline, or the presence of AD pathology cannot be ruled out. Although it is speculative, the presence of pathology or covert disease could potentially have explained the few HC with epileptiform discharges. Overall, we were able to demonstrate in a relatively small sample that an association between spike frequency and normalized rCBF is present in AD, which underlines the neuropathological link between epileptiform discharges and hippocampal rCBF.

## Conclusions

As hypothesized, increasing spike frequency was associated with increasing rCBF in the hippocampus in patients with AD while this could not be shown in HC. Surprisingly, no significant difference in the hippocampal rCBF was found between AD and HC, which may be due to an increased frequency of epileptiform discharges in patients with AD increasing rCBF. The hemodynamic coupling between epileptiform discharges and blood flow has previously been found in patients with epilepsy and may be a shared mechanism across pathologies. Our findings indicate that hyperperfusion may be an accompanying phenomenon to hyperexcitability in patients with AD, which is in support of the theory of a hyperexcitable hippocampus. However, the current study has some limitations and more studies investigating the association between rCBF and epileptiform discharges in AD are needed.

### Electronic supplementary material

Below is the link to the electronic supplementary material.

Supplementary Material 1

## Data Availability

The datasets generated and/or analyzed during the current study are not publicly available due Danish data protection regulations but are available from the corresponding author on reasonable request.
