# Supplementary material for: Subclinical epileptiform discharges in Alzheimer’s disease are associated with increased hippocampal blood flow
Source: Alzheimers Res Ther. 2024 Apr 12;16:80. doi: 10.1186/s13195-024-01432-9 (PMC11010418; doi:10.1186/s13195-024-01432-9)

**Supplementary materials to: “Subclinical epileptiform discharges in Alzheimer’s disease are associated with increased hippocampal blood flow”**

Page 2 – **Supplementary figure 1**

Page 3 – **Supplementary figure 2**

Page 4-7 – **Checking of the linear regression including supplementary figure 3 and 4**

Page 8 – **Supplementary figure 5**

Page 9-10 – **Ear-EEG equipment and pre-processing**

Page 11 **– Supplementary figure 6**

Page 12 – **Supplementary figure 7**

Page 13-50 – **R output**

**
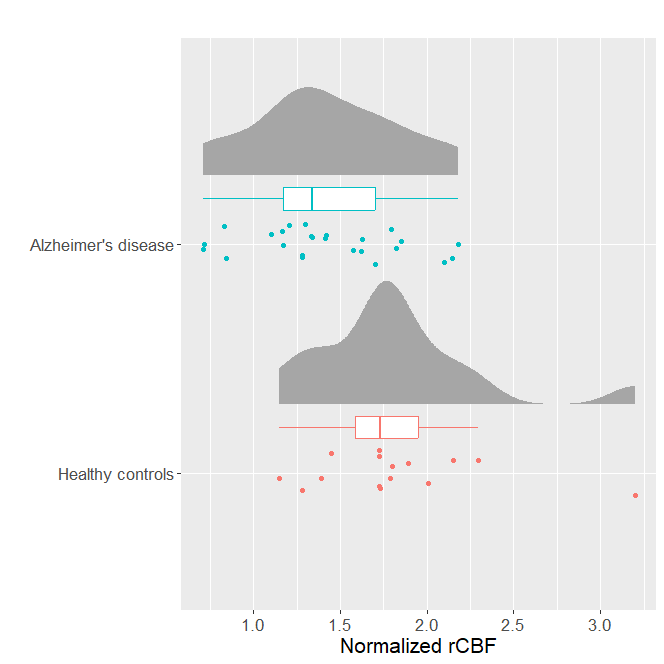
**

**Supplementary figure 1:** Normalized rCBF in the precuneus for HC and AD.


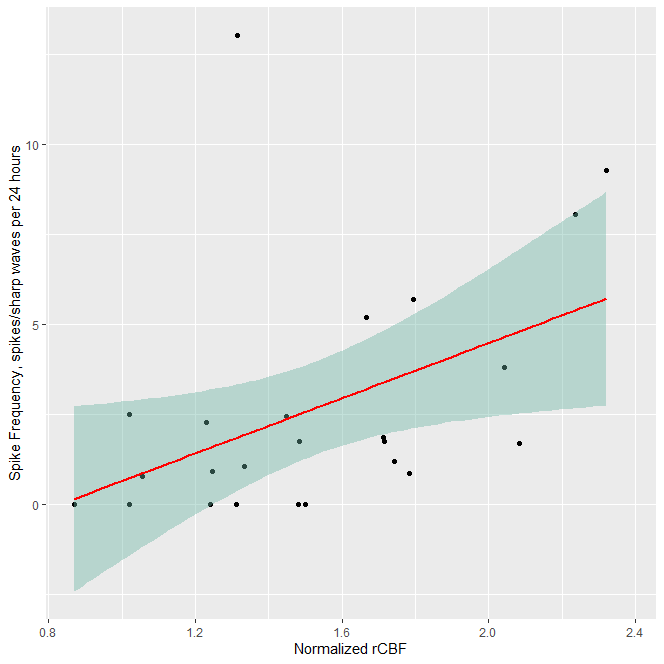


**Supplementary figure 2**: Plot showing the number of spikes/24 hours of recording and the normalized rCBF in the hippocampus in patients with AD. The outlier (marked with red circle) was removed from the subsequent analyses.

**Checking of the linear regression**

A full description of the statistical analysis can found in the following pages including a QQ-plot of the residuals. Here, we wanted to fully understand whether Figure 3 was affected by outliers after removing the most pronounced outlier (Supplementary figure 2). We therefore performed Cook’s distance as follows in R:

library(ggplot2)

cooksd <- cooks.distance(model1_AD)

cooksd_df <- data.frame(observation = seq_along(cooksd), cooksd = cooksd)

n <- nrow(cooksd_df)

k <- length(coefficients(model1_AD)) - 1

threshold <- 4 / (n - k - 1)

ggplot(cooksd_df, aes(x = observation, y = cooksd)) +

geom_point() +

geom_hline(yintercept = threshold, color = "red", linetype = "solid") +

labs(x = "Observation number", y = "Cook's Distance") +

theme_minimal()

Here, we found that none of the remaining data points were substantially higher or exceeded the threshold set at 4/(n - k – 1) where n is the sample size and k is the number of independent variables (see Supplementary figure 3).


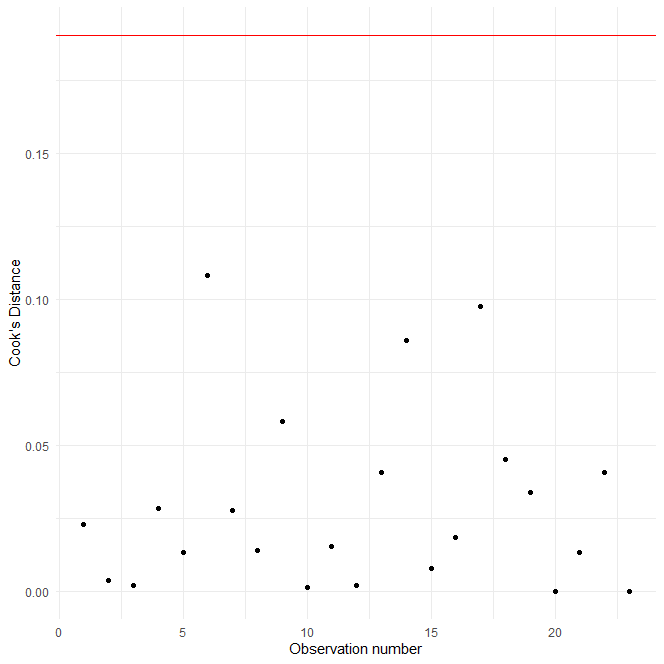


**Supplementary figure 3**: Figure showing the Cook’s distance for each observation and the calculated threshold (red line at 0.19). None of the observations were considered outliers.

However, to understand if the linear regression was affected by the two individuals with high spike frequency, we conducted an additional analysis excluding these individuals as follows:

AD <- AD[-5,]

AD <- AD[-3,]

model1_AD <- lm (AD$Mean_hip_norm~AD$SpikeFrequency)

summary(model1_AD)

plot(model1_AD)

ggplot(AD, aes(x=SpikeFrequency, y=Mean_hip_norm)) +

geom_point() +

geom_smooth(method=lm , color="red", fill="#69b3a2", se=TRUE) +

xlab("Spike Frequency, spikes/sharp waves per 24 hours") + ylab("Normalized rCBF")

ggsave("figure4_high_res.tiff", device='tiff', dpi=300)

Here, we found that the linear regression was still significant (see Supplementary figure 4) with a slightly lower estimate (estimate: 0.09833, p-value: 0.0281.


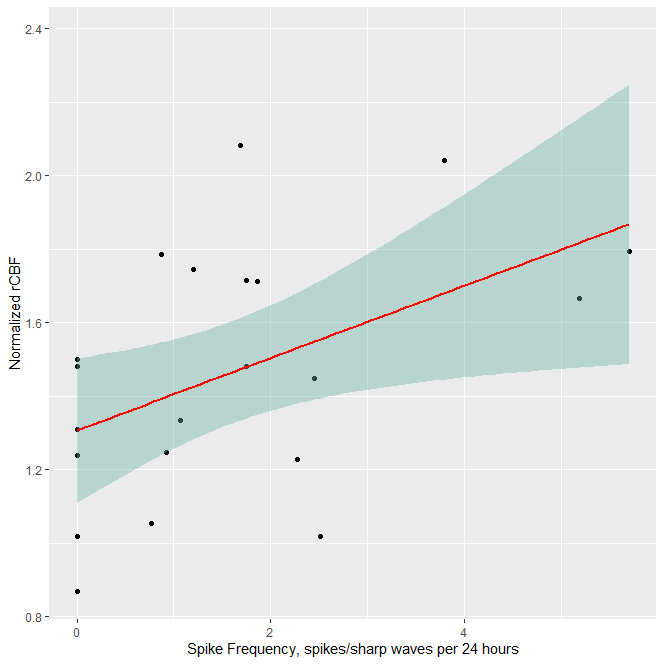


**Supplementary figure 4**: Plot showing the number of spikes/24 hours of recording and the normalized rCBF in the hippocampus in patients with AD. The outlier (as marked in Supplementary figure 3) as well as the two observations with high spike frequency were removed from the analyses.

**
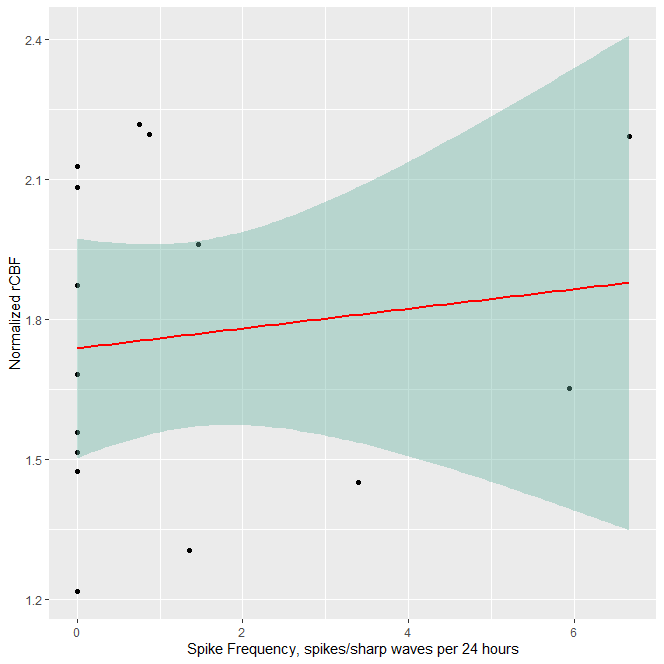
**

**Supplementary figure 5**: Plot showing the number of spikes/24 hours of recording and the normalized rCBF in the hippocampus in patients with HC. No significant was found (estimate: 0.021, t-value = 0.491, p-value = 0.632).

**Ear-EEG equipment and pre-processing**

The ear-EEG earpieces were custom-made in silicone and mounted with six dry-electrodes [1] (T&W engineering, Denmark). The ear-piece was placed inside the ear canal and in the concha [2] in both ears with the labeling of the ear-EEG electrodes being previously described [3]. The ground electrode was placed approximately 2 cm under the midline of the clavicle on the left or right side. The ear-EEG recordings were performed using the TMSi Mobita EEG amplifier (TMSi systems), which was worn in either a small bag around the neck or in a belt bag and connected to the electrodes. The sampling rate was 1000 Hz. The EEG data were loaded into MATLAB (MathWorks, v2020b) and the EEGLAB [4] data structure was used throughout the analysis. See feasibility study for a complete description of the pre-processing [5].

Ear-EEG recordings were visually reviewed and annotated by CSM using the EEGLAB toolbox (v13.6.5b) implemented in MATLAB. The average referenced montage was used and 10 second epochs were inspected at a time with ±30 µV amplitude range. In this montage, the first 12 channels were low-pass filtered at 70 Hz, high-pass filtered at 1 Hz, and notch filtered at 50 Hz. The next 12 channels were after pre-processing, which included removal of segments with excessive artifacts. The subsequent 36 channels were the comparisons between electrodes with the active electrodes being on the left (e.g., ELA-ERA, ELA-ERB [3]) and the last 36 channels were created using the opposite laterality. The segments disregarded by the pre-processing pipeline and segments only containing recordings from one ear were not inspected.

References

[1] Kappel SL, Rank ML, Toft HO, Andersen M, Kidmose P (2019) Dry-Contact Electrode Ear-EEG. *IEEE Trans Biomed Eng* **66**, 150–158.

[2] Mikkelsen KB, Kappel SL, Mandic DP, Kidmose P (2015) EEG Recorded from the Ear: Characterizing the Ear-EEG Method. *Front Neurosci* **9**,.

[3] Kidmose P, Looney D, Mandic DP (2012) Auditory evoked responses from Ear-EEG recordings. In *2012 Annual International Conference of the IEEE engineering in Medicine and Biology Society* IEEE.

[4] Delorme A, Makeig S (2004) EEGLAB: an open source toolbox for analysis of single-trial EEG dynamics including independent component analysis. *J Neurosci Methods* **134**, 9–21.

[5] Musaeus CS, Waldemar G, Andersen BB, Høgh P, Kidmose P, Hemmsen MC, Rank ML, Kjær TW, Frederiksen KS (2022) Long-Term EEG Monitoring in Patients with Alzheimer’s Disease Using Ear-EEG: A Feasibility Study. *J Alzheimer’s Dis* **In Press**,.

**
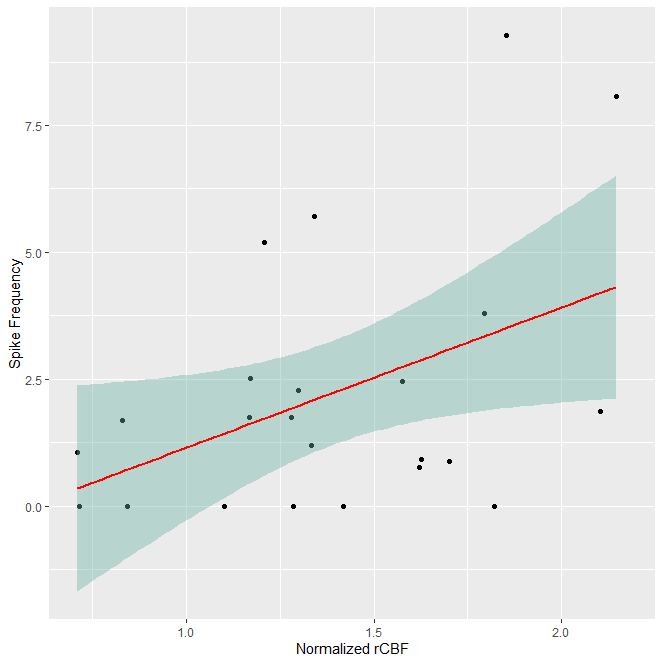
**

**Supplementary figure 6**: Figure showing the linear association between spike frequency and normalized rCBF in precuneus in patients with AD

**
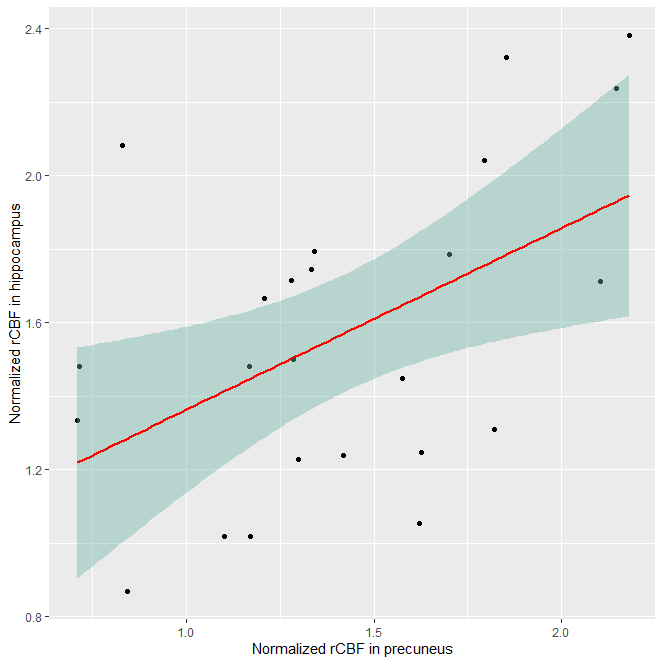
**

**Supplementary figure 7:** The association between normalized rCBF in precuneus and normalized rCBF in the hippocampus in patients with AD

Subclinical epileptiform discharges in Alzheimer’s disease are associated with increased hippocampal blood flow

Musaeus CS et al.

15/3/2023

## Demographics
Demogra$Diagnosis <- as.factor(Demogra$Diagnosis)

#Subset
AD <- subset(Demogra, Diagnosis == "Alzheimer's disease")
HC <- subset(Demogra, Diagnosis == "Healthy Control")

# Age
qqnorm(AD$Age)
qqline(AD$Age)


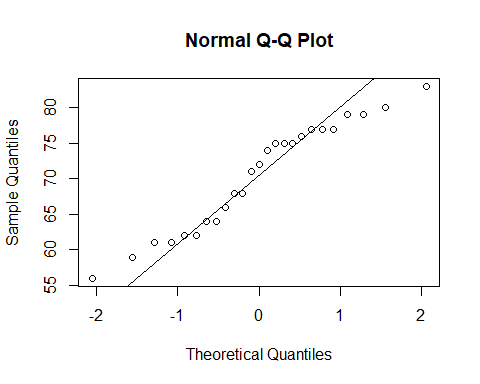


qqnorm(HC$Age)
qqline(HC$Age)


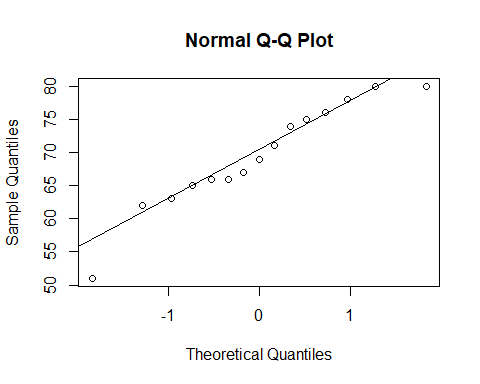


var.test(AD$Age, HC$Age)

##
## F test to compare two variances
##
## data: AD$Age and HC$Age
## F = 0.93372, num df = 24, denom df = 14, p-value = 0.8535
## alternative hypothesis: true ratio of variances is not equal to 1
## 95 percent confidence interval:
## 0.3348101 2.3041107
## sample estimates:
## ratio of variances
## 0.9337223

t.test(AD$Age, HC$Age, var.equal = TRUE)

##
## Two Sample t-test
##
## data: AD$Age and HC$Age
## t = 0.35777, df = 38, p-value = 0.7225
## alternative hypothesis: true difference in means is not equal to 0
## 95 percent confidence interval:
## -4.223548 6.036881
## sample estimates:
## mean of x mean of y
## 70.44000 69.53333

# Gender
Gender_diagnosis <- data.frame(G1=c(as.numeric(8),as.numeric(7)),
 G2=c(as.numeric(15),as.numeric(10)))


chisq.test(Gender_diagnosis, correct = F)

##
## Pearson's Chi-squared test
##
## data: Gender_diagnosis
## X-squared = 0.1705, df = 1, p-value = 0.6797

# Education

qqnorm(AD$Education)
qqline(AD$Education)


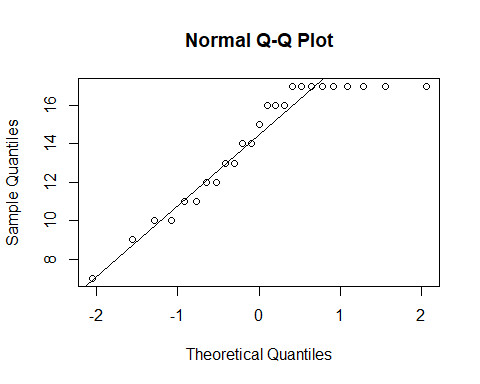


qqnorm(HC$Education)
qqline(HC$Education)


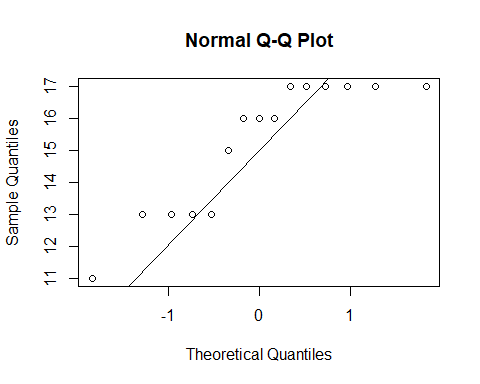


var.test(AD$Education, HC$Education)

##
## F test to compare two variances
##
## data: AD$Education and HC$Education
## F = 2.2758, num df = 24, denom df = 14, p-value = 0.1127
## alternative hypothesis: true ratio of variances is not equal to 1
## 95 percent confidence interval:
## 0.8160463 5.6159018
## sample estimates:
## ratio of variances
## 2.275799

t.test(AD$Education, HC$Education, var.equal = TRUE)

##
## Two Sample t-test
##
## data: AD$Education and HC$Education
## t = -1.2495, df = 38, p-value = 0.2191
## alternative hypothesis: true difference in means is not equal to 0
## 95 percent confidence interval:
## -2.9346079 0.6946079
## sample estimates:
## mean of x mean of y
## 14.08 15.20

# MMSE

qqnorm(AD$MMSE_1)
qqline(AD$MMSE_1)


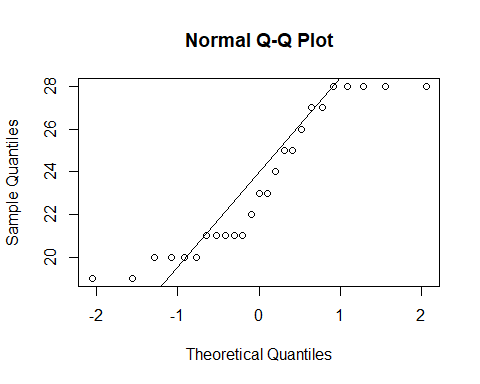


qqnorm(HC$MMSE_1)
qqline(HC$MMSE_1)


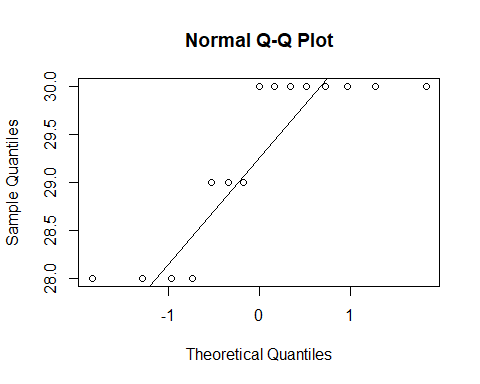


var.test(AD$MMSE_1, HC$MMSE_1)

##
## F test to compare two variances
##
## data: AD$MMSE_1 and HC$MMSE_1
## F = 13.872, num df = 24, denom df = 14, p-value = 7.478e-06
## alternative hypothesis: true ratio of variances is not equal to 1
## 95 percent confidence interval:
## 4.974145 34.231280
## sample estimates:
## ratio of variances
## 13.87195

t.test(AD$MMSE_1, HC$MMSE_1, var.equal = FALSE)

##
## Welch Two Sample t-test
##
## data: AD$MMSE_1 and HC$MMSE_1
## t = -8.4206, df = 29.386, p-value = 2.515e-09
## alternative hypothesis: true difference in means is not equal to 0
## 95 percent confidence interval:
## -7.290774 -4.442560
## sample estimates:
## mean of x mean of y
## 23.40000 29.26667

tid_dif <- read_excel("Tid_dif.xlsx") #
tid_dif$Diagnosis <- as.factor(tid_dif$Diagnosis)

tid_dif <- tid_dif[-17,] # Remove patient without ear-EEG

AD_tiddif <- subset(tid_dif, Diagnosis == "2")
HC_tiddif <- subset(tid_dif, Diagnosis == "1")


qqnorm(AD_tiddif$Tid_dif)
qqline(AD_tiddif$Tid_dif)


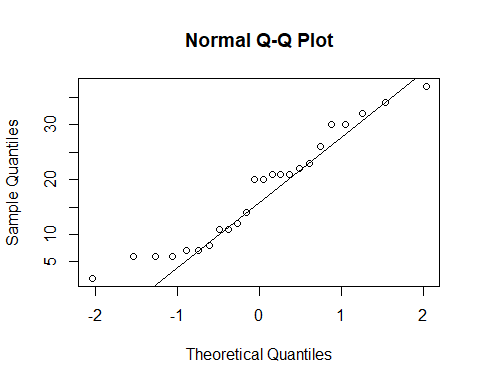


qqnorm(HC_tiddif$Tid_dif)
qqline(HC_tiddif$Tid_dif)


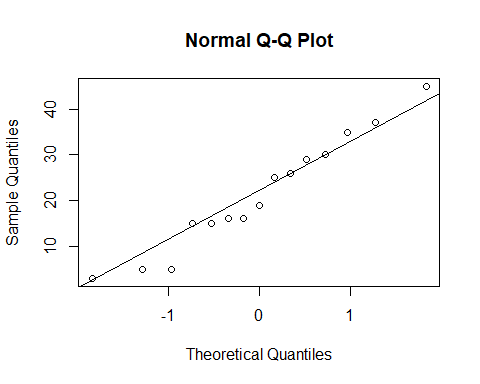


var.test(AD_tiddif$Tid_dif, HC_tiddif$Tid_dif)

##
## F test to compare two variances
##
## data: AD_tiddif$Tid_dif and HC_tiddif$Tid_dif
## F = 0.67769, num df = 23, denom df = 14, p-value = 0.3954
## alternative hypothesis: true ratio of variances is not equal to 1
## 95 percent confidence interval:
## 0.2419556 1.6919153
## sample estimates:
## ratio of variances
## 0.6776859

t.test(AD_tiddif$Tid_dif, HC_tiddif$Tid_dif, var.equal = TRUE)

##
## Two Sample t-test
##
## data: AD_tiddif$Tid_dif and HC_tiddif$Tid_dif
## t = -0.9839, df = 37, p-value = 0.3316
## alternative hypothesis: true difference in means is not equal to 0
## 95 percent confidence interval:
## -11.039132 3.822466
## sample estimates:
## mean of x mean of y
## 17.79167 21.40000

AD_tiddif_both <- subset(tid_dif, Diagnosis == "2" | Diagnosis == "1")
mean(AD_tiddif_both$Tid_dif)

## [1] 19.17949

#
#
## Structural ##
#
#


Hippo$Diagnose <- as.factor(Hippo$Diagnose)
Hippo$Gender <- as.factor(Hippo$Gender)

Hippo$Left_hippo_c <- Hippo$Left_hippo/Hippo$Total_vol*100
Hippo$Right_hippo_c <- Hippo$Right_hippo/Hippo$Total_vol*100

AD_hippo <- subset(Hippo, Diagnose == "2")
HC_hippo <- subset(Hippo, Diagnose == "1")

# Left
qqnorm(AD_hippo$Left_hippo_c)
qqline(AD_hippo$Left_hippo_c)


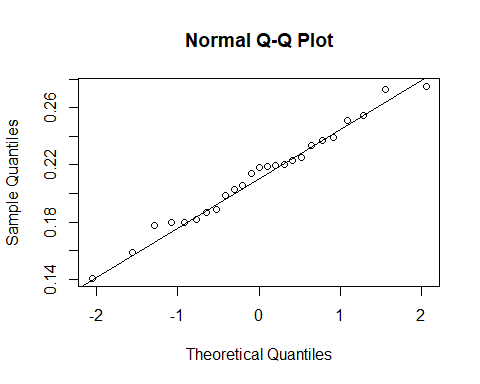


qqnorm(HC_hippo$Left_hippo_c)
qqline(HC_hippo$Left_hippo_c)


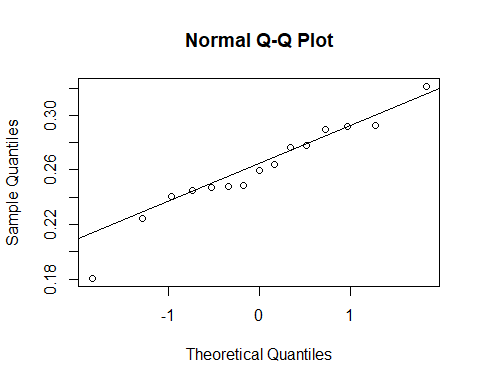


var.test(AD_hippo$Left_hippo_c, HC_hippo$Left_hippo_c)

##
## F test to compare two variances
##
## data: AD_hippo$Left_hippo_c and HC_hippo$Left_hippo_c
## F = 0.98454, num df = 24, denom df = 14, p-value = 0.9407
## alternative hypothesis: true ratio of variances is not equal to 1
## 95 percent confidence interval:
## 0.3530307 2.4295019
## sample estimates:
## ratio of variances
## 0.9845361

t.test(AD_hippo$Left_hippo_c, HC_hippo$Left_hippo_c, var.equal = TRUE)

##
## Two Sample t-test
##
## data: AD_hippo$Left_hippo_c and HC_hippo$Left_hippo_c
## t = -4.3958, df = 38, p-value = 8.584e-05
## alternative hypothesis: true difference in means is not equal to 0
## 95 percent confidence interval:
## -0.07045393 -0.02602313
## sample estimates:
## mean of x mean of y
## 0.2122014 0.2604400

#Right
qqnorm(AD_hippo$Right_hippo_c)
qqline(AD_hippo$Right_hippo_c)


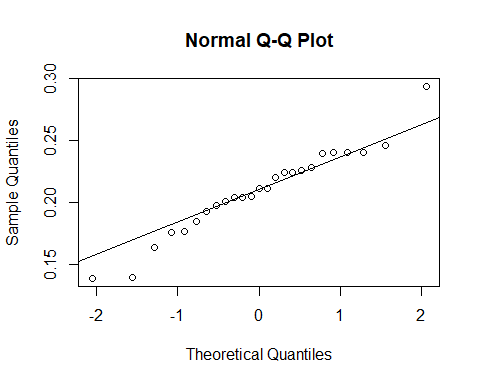


qqnorm(HC_hippo$Right_hippo_c)
qqline(HC_hippo$Right_hippo_c)


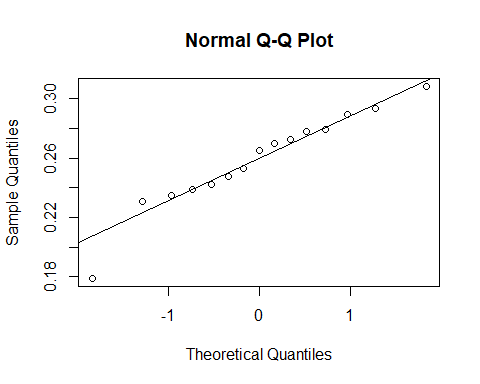


var.test(AD_hippo$Right_hippo_c, HC_hippo$Right_hippo_c)

##
## F test to compare two variances
##
## data: AD_hippo$Right_hippo_c and HC_hippo$Right_hippo_c
## F = 1.1758, num df = 24, denom df = 14, p-value = 0.7708
## alternative hypothesis: true ratio of variances is not equal to 1
## 95 percent confidence interval:
## 0.4215992 2.9013790
## sample estimates:
## ratio of variances
## 1.175761

t.test(AD_hippo$Right_hippo_c, HC_hippo$Right_hippo_c, var.equal = TRUE)

##
## Two Sample t-test
##
## data: AD_hippo$Right_hippo_c and HC_hippo$Right_hippo_c
## t = -4.5053, df = 38, p-value = 6.142e-05
## alternative hypothesis: true difference in means is not equal to 0
## 95 percent confidence interval:
## -0.07177459 -0.02727045
## sample estimates:
## mean of x mean of y
## 0.2092538 0.2587763

#
#
# Flow
#
#

Flow <- read_excel("Flow.xlsx") #
Flow$Diagnosis <- as.factor(Flow$Diagnosis)
Flow$Gender <- as.factor(Flow$Gender)

AD_Flow <- subset(Flow, Diagnosis == "2")
HC_Flow <- subset(Flow, Diagnosis == "1")

# Flow
qqnorm(AD_Flow$Flow)
qqline(AD_Flow$Flow) # Two outliers


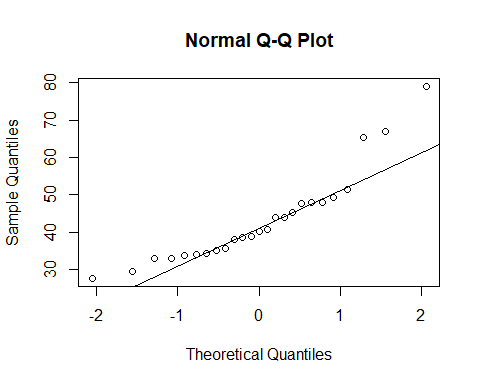


qqnorm(HC_Flow$Flow)
qqline(HC_Flow$Flow)


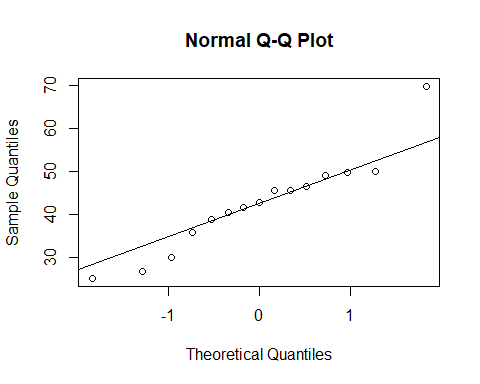


var.test(AD_Flow$Flow, HC_Flow$Flow)

##
## F test to compare two variances
##
## data: AD_Flow$Flow and HC_Flow$Flow
## F = 1.2441, num df = 24, denom df = 14, p-value = 0.6845
## alternative hypothesis: true ratio of variances is not equal to 1
## 95 percent confidence interval:
## 0.4460962 3.0699637
## sample estimates:
## ratio of variances
## 1.244078

t.test(AD_Flow$Flow, HC_Flow$Flow, var.equal = TRUE)

##
## Two Sample t-test
##
## data: AD_Flow$Flow and HC_Flow$Flow
## t = 0.16231, df = 38, p-value = 0.8719
## alternative hypothesis: true difference in means is not equal to 0
## 95 percent confidence interval:
## -7.190683 8.444229
## sample estimates:
## mean of x mean of y
## 43.22720 42.60043

#
#
# ASL
#
#

ASL$Diagnosis <- as.factor(ASL$Diagnosis)

ASL$Mean_hippo_c <- (Hippo$Left_hippo+Hippo$Right_hippo)/Hippo$Total_vol*100

ASL$Left_hip_norm <- ASL$Left_hip/ASL$Flow
ASL$Right_hip_norm <- ASL$Right_hip/ASL$Flow
ASL$Mean_hip_norm <- (ASL$Mean_hip)/ASL$Flow

AD_ASL <- subset(ASL, Diagnosis =="2")
HC_ASL <- subset(ASL, Diagnosis =="1")

# Left hippocampus
qqnorm(AD_ASL$Left_hip_norm)
qqline(AD_ASL$Left_hip_norm)


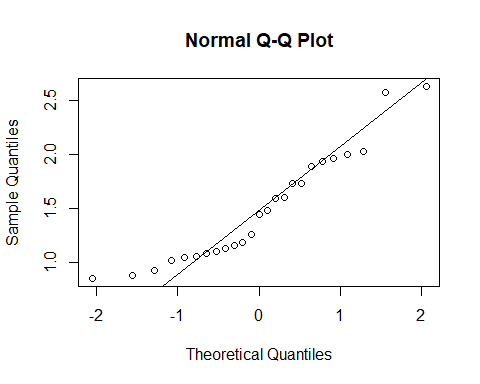


qqnorm(HC_ASL$Left_hip_norm)
qqline(HC_ASL$Left_hip_norm)


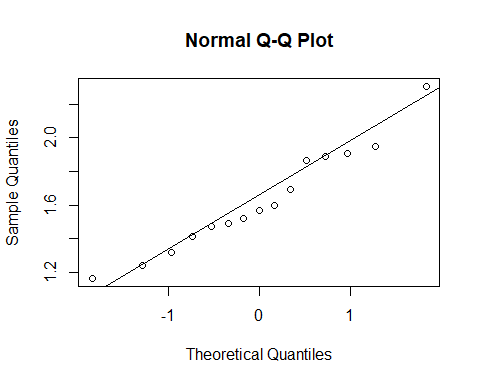


var.test(AD_ASL$Left_hip_norm, HC_ASL$Left_hip_norm)

##
## F test to compare two variances
##
## data: AD_ASL$Left_hip_norm and HC_ASL$Left_hip_norm
## F = 2.6925, num df = 24, denom df = 14, p-value = 0.05793
## alternative hypothesis: true ratio of variances is not equal to 1
## 95 percent confidence interval:
## 0.9654724 6.6442287
## sample estimates:
## ratio of variances
## 2.69252

t.test(AD_ASL$Left_hip_norm, HC_ASL$Left_hip_norm, var.equal = TRUE)

##
## Two Sample t-test
##
## data: AD_ASL$Left_hip_norm and HC_ASL$Left_hip_norm
## t = -0.91265, df = 38, p-value = 0.3672
## alternative hypothesis: true difference in means is not equal to 0
## 95 percent confidence interval:
## -0.4258929 0.1612105
## sample estimates:
## mean of x mean of y
## 1.494728 1.627070

# Right Hippocampus
qqnorm(AD_ASL$Right_hip_norm)
qqline(AD_ASL$Right_hip_norm)


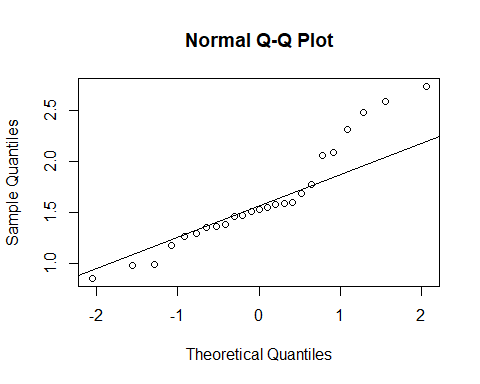


qqnorm(HC_ASL$Right_hip_norm)
qqline(HC_ASL$Right_hip_norm)


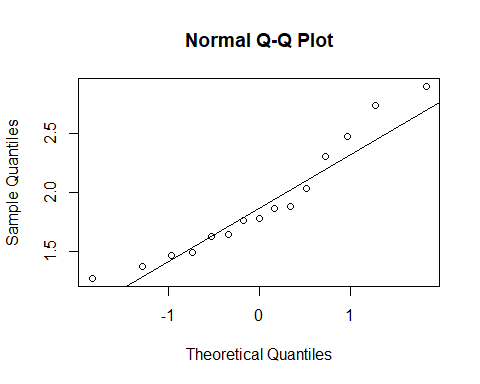


var.test(AD_ASL$Right_hip_norm, HC_ASL$Right_hip_norm)

##
## F test to compare two variances
##
## data: AD_ASL$Right_hip_norm and HC_ASL$Right_hip_norm
## F = 1.0064, num df = 24, denom df = 14, p-value = 0.977
## alternative hypothesis: true ratio of variances is not equal to 1
## 95 percent confidence interval:
## 0.3608717 2.4834620
## sample estimates:
## ratio of variances
## 1.006403

t.test(AD_ASL$Right_hip_norm, HC_ASL$Right_hip_norm, var.equal = TRUE)

##
## Two Sample t-test
##
## data: AD_ASL$Right_hip_norm and HC_ASL$Right_hip_norm
## t = -1.7302, df = 38, p-value = 0.09172
## alternative hypothesis: true difference in means is not equal to 0
## 95 percent confidence interval:
## -0.60918590 0.04774172
## sample estimates:
## mean of x mean of y
## 1.627635 1.908357

# Mean hippocampus
qqnorm(AD_ASL$Mean_hip_norm)
qqline(AD_ASL$Mean_hip_norm)


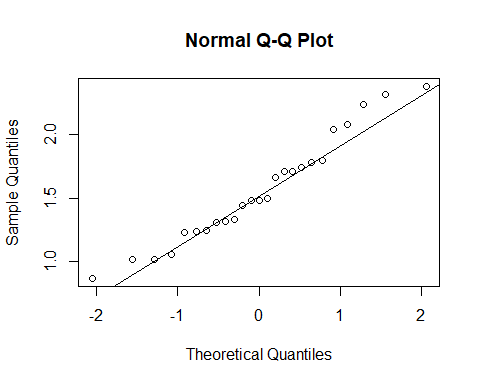


qqnorm(HC_ASL$Mean_hip_norm)
qqline(HC_ASL$Mean_hip_norm)

var.test(AD_ASL$Mean_hip_norm, HC_ASL$Mean_hip_norm)

##
## F test to compare two variances
##
## data: AD_ASL$Mean_hip_norm and HC_ASL$Mean_hip_norm
## F = 1.4764, num df = 24, denom df = 14, p-value = 0.453
## alternative hypothesis: true ratio of variances is not equal to 1
## 95 percent confidence interval:
## 0.5294086 3.6433067
## sample estimates:
## ratio of variances
## 1.476421

t.test(AD_ASL$Mean_hip_norm, HC_ASL$Mean_hip_norm, var.equal = TRUE)

##
## Two Sample t-test
##
## data: AD_ASL$Mean_hip_norm and HC_ASL$Mean_hip_norm
## t = -1.6014, df = 38, p-value = 0.1176
## alternative hypothesis: true difference in means is not equal to 0
## 95 percent confidence interval:
## -0.46762018 0.05455832
## sample estimates:
## mean of x mean of y
## 1.561178 1.767709

library(tidyverse)

## -- Attaching packages --------------------------------------- tidyverse 1.3.2 --
## v ggplot2 3.4.0 v purrr 0.3.4
## v tibble 3.1.7 v dplyr 1.0.9
## v tidyr 1.2.0 v stringr 1.4.0
## v readr 2.1.2 v forcats 0.5.2
## -- Conflicts ------------------------------------------ tidyverse_conflicts() --
## x dplyr::filter() masks stats::filter()
## x dplyr::lag() masks stats::lag()


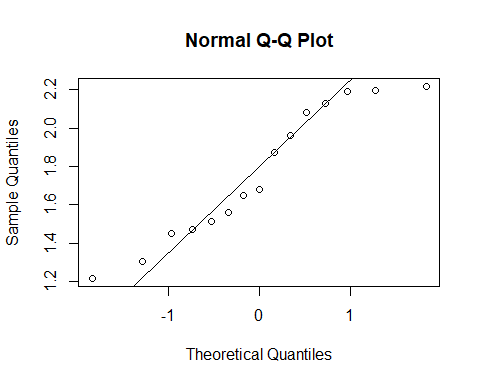


library(ggdist)
library(ggplot2)

ASL_plot <- subset(ASL, Diagnosis == "1" | Diagnosis == "2")

ggplot(ASL_plot, aes(x = Mean_hip_norm, y = Diagnosis, color = Diagnosis)) +
 stat_halfeye(
 point_color = NA, .width = 0, height = 0.6,
 position = position_nudge(y = 0.3)
 ) +
 geom_boxplot(
 position = position_nudge(y = 0.2),
 width = 0.1, outlier.shape = NA
 ) +
 geom_point(position = position_jitter(width = 0, height = 0.1, seed = 1)) +
 ggtitle("") + xlab("Normalized rCBF") + ylab("") +scale_y_discrete(breaks=c("1","2"),labels=c("Healthy controls", "Alzheimer's disease")) + theme(legend.position = "none") +
 theme(text = element_text(size = 16))


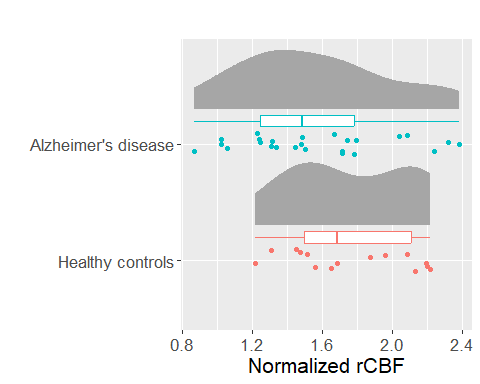


#
#
# Plot Linear regression
#
#

AD <- subset(ASL, Diagnosis == "2")

model1_AD <- lm (AD$Mean_hip_norm~AD$SpikeFrequency)
summary(model1_AD)

##
## Call:
## lm(formula = AD$Mean_hip_norm ~ AD$SpikeFrequency)
##
## Residuals:
## Min 1Q Median 3Q Max
## -0.7578 -0.2100 0.0053 0.2512 0.6079
##
## Coefficients:
## Estimate Std. Error t value Pr(>|t|)
## (Intercept) 1.38625 0.09449 14.672 7.66e-13 ***
## AD$SpikeFrequency 0.05263 0.02233 2.356 0.0278 *
## ---
## Signif. codes: 0 '***' 0.001 '**' 0.01 '*' 0.05 '.' 0.1 ' ' 1
##
## Residual standard error: 0.3587 on 22 degrees of freedom
## (1 observation deleted due to missingness)
## Multiple R-squared: 0.2015, Adjusted R-squared: 0.1652
## F-statistic: 5.552 on 1 and 22 DF, p-value: 0.02778

plot(model1_AD)


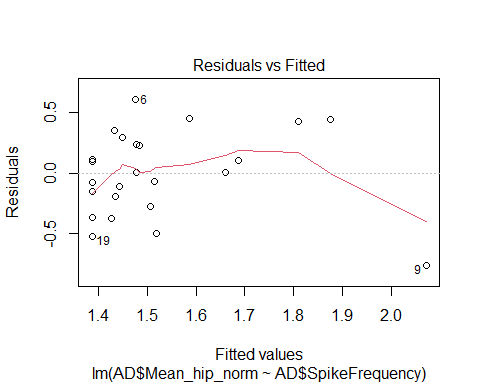

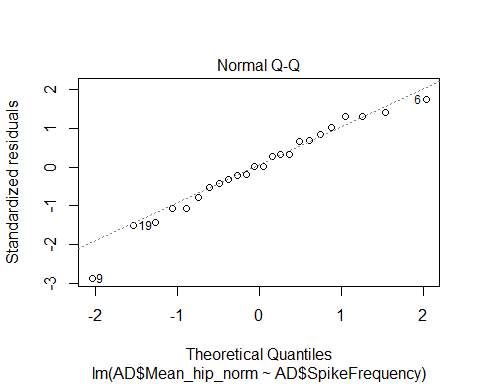

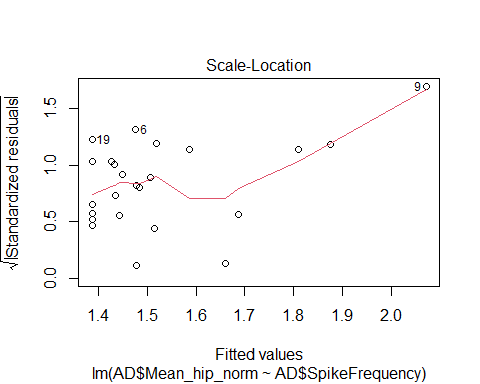

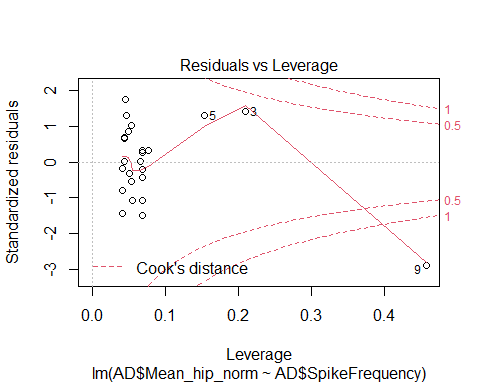


AD <- AD[-9,]

model1_AD <- lm (AD$Mean_hip_norm~AD$SpikeFrequency)
summary(model1_AD)

##
## Call:
## lm(formula = AD$Mean_hip_norm ~ AD$SpikeFrequency)
##
## Residuals:
## Min 1Q Median 3Q Max
## -0.54794 -0.17011 -0.00172 0.21061 0.60487
##
## Coefficients:
## Estimate Std. Error t value Pr(>|t|)
## (Intercept) 1.29437 0.08078 16.024 2.99e-13 ***
## AD$SpikeFrequency 0.10877 0.02404 4.524 0.000186 ***
## ---
## Signif. codes: 0 '***' 0.001 '**' 0.01 '*' 0.05 '.' 0.1 ' ' 1
##
## Residual standard error: 0.2904 on 21 degrees of freedom
## (1 observation deleted due to missingness)
## Multiple R-squared: 0.4936, Adjusted R-squared: 0.4695
## F-statistic: 20.47 on 1 and 21 DF, p-value: 0.0001857

plot(model1_AD)


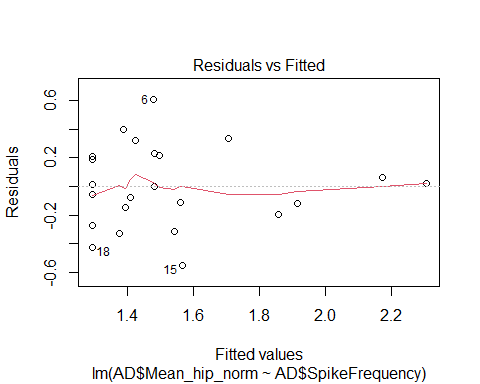

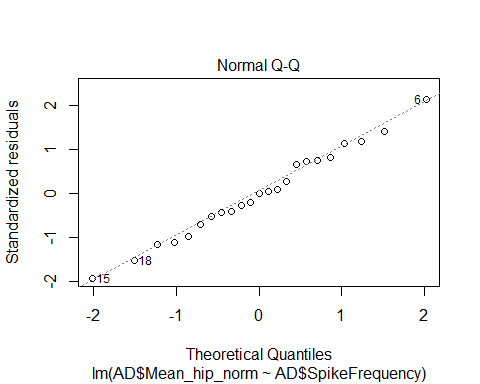

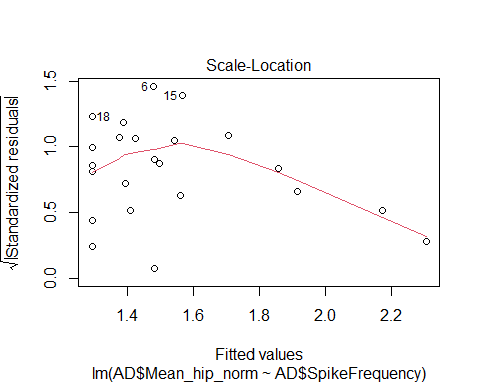

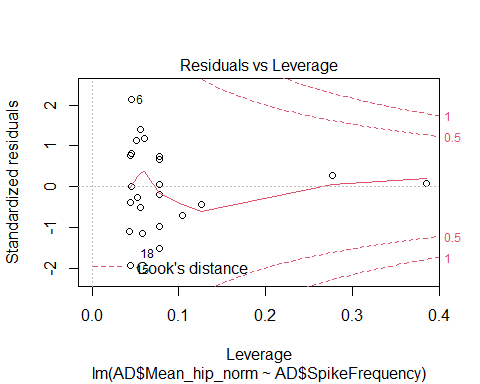


ggplot(AD, aes(x=Mean_hip_norm, y=SpikeFrequency)) +
 geom_point() +
 geom_smooth(method=lm , color="red", fill="#69b3a2", se=TRUE) +
 xlab("Normalized rCBF") + ylab("Spike Frequency, spikes/sharp waves per 24 hours")

## `geom_smooth()` using formula = 'y ~ x'

## Warning: Removed 1 rows containing non-finite values (`stat_smooth()`).

## Warning: Removed 1 rows containing missing values (`geom_point()`).


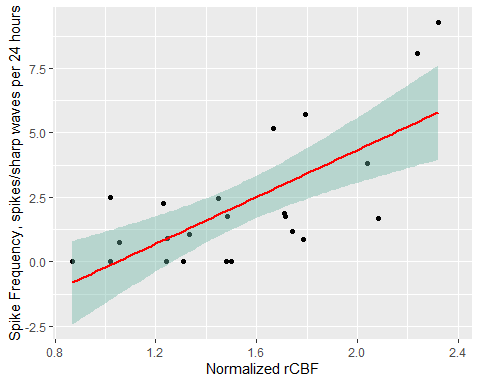


# Precuneus


Precuneus <- read.delim("MyFile_precuneus.txt", sep = ",", dec = ".")

Precuneus$Diagnosis <- Flow$Diagnosis

Precuneus$Diagnosis <- as.factor(Precuneus$Diagnosis)

Precuneus$Precuneus_norm <- Precuneus$Precuneus/Precuneus$Flow
Precuneus$gender <- as.factor(Precuneus$gender)

AD_precuneus <- subset(Precuneus, Diagnosis == "2")
HC_precuneus <- subset(Precuneus, Diagnosis == "1")

# Precuneus
qqnorm(AD_precuneus$Precuneus_norm)
qqline(AD_precuneus$Precuneus_norm)


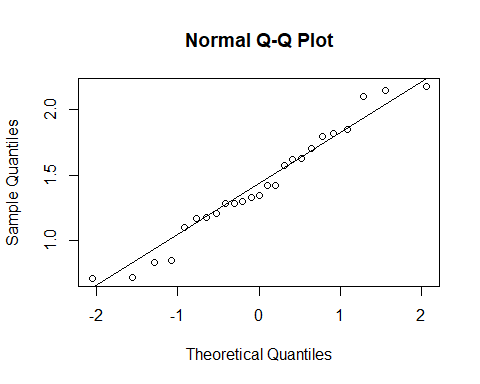


qqnorm(HC_precuneus$Precuneus_norm)
qqline(HC_precuneus$Precuneus_norm)


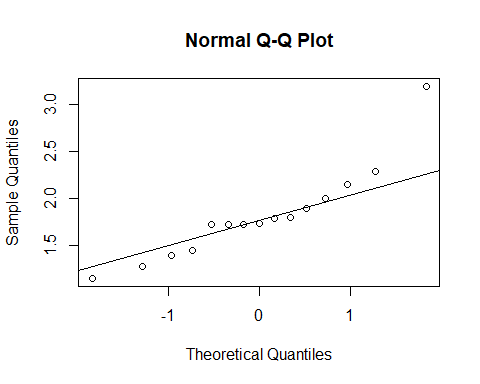


var.test(AD_precuneus$Precuneus_norm, HC_precuneus$Precuneus_norm)

##
## F test to compare two variances
##
## data: AD_precuneus$Precuneus_norm and HC_precuneus$Precuneus_norm
## F = 0.73881, num df = 24, denom df = 14, p-value = 0.4983
## alternative hypothesis: true ratio of variances is not equal to 1
## 95 percent confidence interval:
## 0.2649192 1.8231320
## sample estimates:
## ratio of variances
## 0.7388096

t.test(AD_precuneus$Precuneus_norm, HC_precuneus$Precuneus_norm, var.equal = TRUE)

##
## Two Sample t-test
##
## data: AD_precuneus$Precuneus_norm and HC_precuneus$Precuneus_norm
## t = -2.7293, df = 38, p-value = 0.009563
## alternative hypothesis: true difference in means is not equal to 0
## 95 percent confidence interval:
## -0.6956283 -0.1031489
## sample estimates:
## mean of x mean of y
## 1.421242 1.820631

ASL_plot_precuneus <- subset(Precuneus, Diagnosis == "1" | Diagnosis == "2")

ggplot(ASL_plot_precuneus, aes(x = Precuneus_norm, y = Diagnosis, color = Diagnosis)) +
 stat_halfeye(
 point_color = NA, .width = 0, height = 0.6,
 position = position_nudge(y = 0.3)
 ) +
 geom_boxplot(
 position = position_nudge(y = 0.2),
 width = 0.1, outlier.shape = NA
 ) +
 geom_point(position = position_jitter(width = 0, height = 0.1, seed = 1)) +
 ggtitle("") + xlab("Normalized rCBF") + ylab("") +scale_y_discrete(breaks=c("1","2"),labels=c("Healthy controls", "Alzheimer's disease")) + theme(legend.position = "none") +
 theme(text = element_text(size = 16))


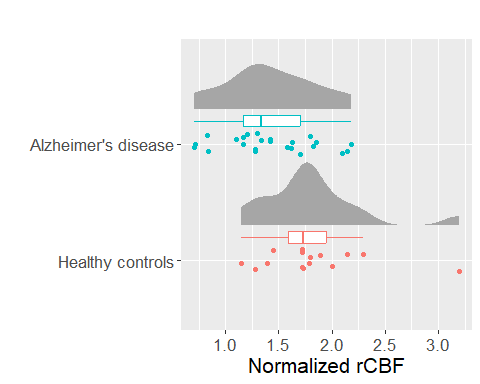


# Association SF and norm rCBF in precuneus
AD_precuneus <- AD_precuneus[-9,]
library(ggplot2)
model1 <- lm (AD_precuneus$Precuneus_norm~AD_precuneus$SpikeFrequency)
summary(model1)

##
## Call:
## lm(formula = AD_precuneus$Precuneus_norm ~ AD_precuneus$SpikeFrequency)
##
## Residuals:
## Min 1Q Median 3Q Max
## -0.59895 -0.26321 -0.02564 0.31213 0.73855
##
## Coefficients:
## Estimate Std. Error t value Pr(>|t|)
## (Intercept) 1.23417 0.10443 11.818 9.65e-11 ***
## AD_precuneus$SpikeFrequency 0.06940 0.03108 2.233 0.0366 *
## ---
## Signif. codes: 0 '***' 0.001 '**' 0.01 '*' 0.05 '.' 0.1 ' ' 1
##
## Residual standard error: 0.3755 on 21 degrees of freedom
## (1 observation deleted due to missingness)
## Multiple R-squared: 0.1919, Adjusted R-squared: 0.1534
## F-statistic: 4.986 on 1 and 21 DF, p-value: 0.03657

plot(model1)


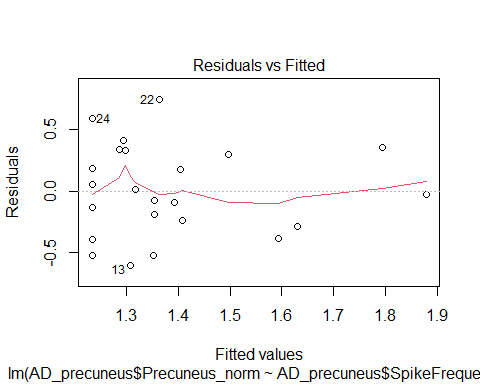

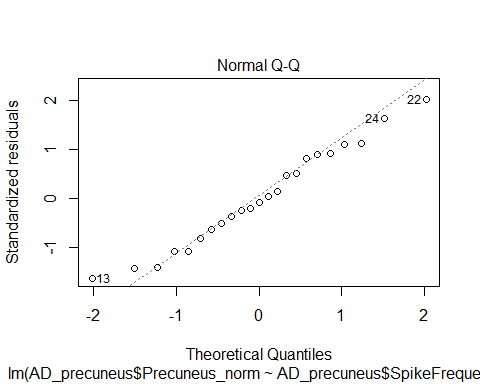

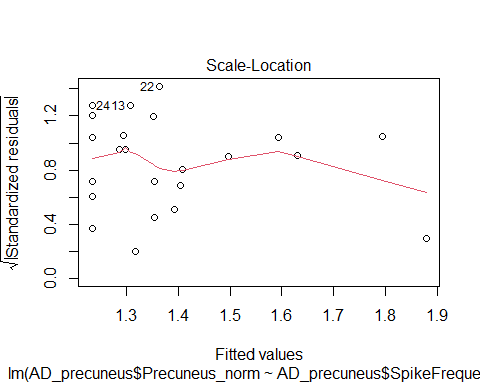

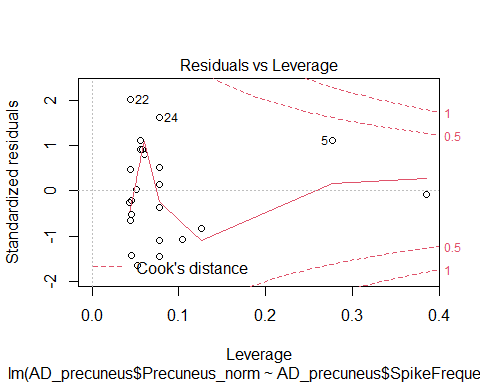


ggplot(AD_precuneus, aes(x=Precuneus_norm, y=SpikeFrequency)) +
 geom_point() +
 geom_smooth(method=lm , color="red", fill="#69b3a2", se=TRUE) +
 xlab("Normalized rCBF") + ylab("Spike Frequency")

## `geom_smooth()` using formula = 'y ~ x'

## Warning: Removed 1 rows containing non-finite values (`stat_smooth()`).
## Removed 1 rows containing missing values (`geom_point()`).


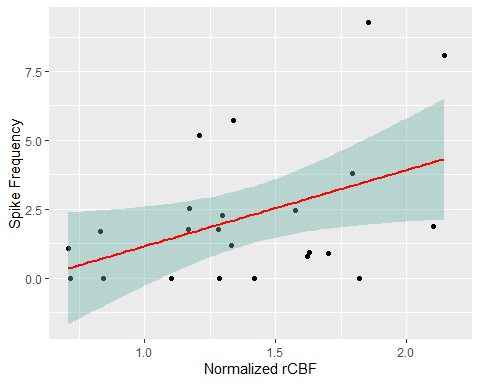


confint(model1)

## 2.5 % 97.5 %
## (Intercept) 1.016983580 1.4513470
## AD_precuneus$SpikeFrequency 0.004764957 0.1340434

# Association Precuneus and Hippocampus in AD
ASL2 = subset(ASL, Diagnosis == "2" )
ASL22 <- ASL2[-9,]
ASL22$precuneus <- AD_precuneus$Precuneus_norm

model1 <- lm (ASL22$precuneus~ASL22$Mean_hip_norm)
summary(model1)

##
## Call:
## lm(formula = ASL22$precuneus ~ ASL22$Mean_hip_norm)
##
## Residuals:
## Min 1Q Median 3Q Max
## -0.84977 -0.21041 0.03865 0.25064 0.60950
##
## Coefficients:
## Estimate Std. Error t value Pr(>|t|)
## (Intercept) 0.6291 0.3031 2.075 0.0498 *
## ASL22$Mean_hip_norm 0.5042 0.1864 2.704 0.0130 *
## ---
## Signif. codes: 0 '***' 0.001 '**' 0.01 '*' 0.05 '.' 0.1 ' ' 1
##
## Residual standard error: 0.3813 on 22 degrees of freedom
## Multiple R-squared: 0.2495, Adjusted R-squared: 0.2154
## F-statistic: 7.313 on 1 and 22 DF, p-value: 0.01295

plot(model1)


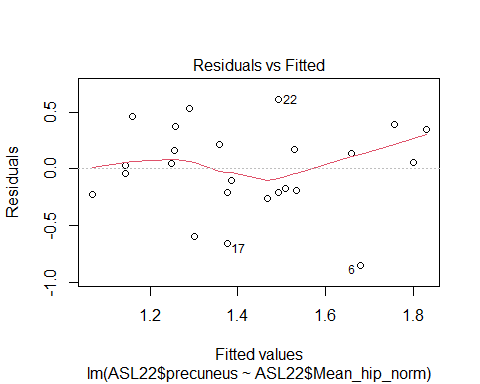

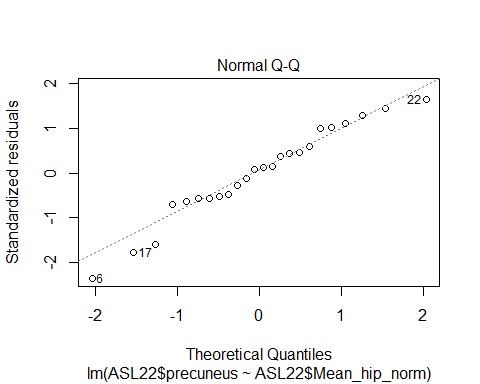

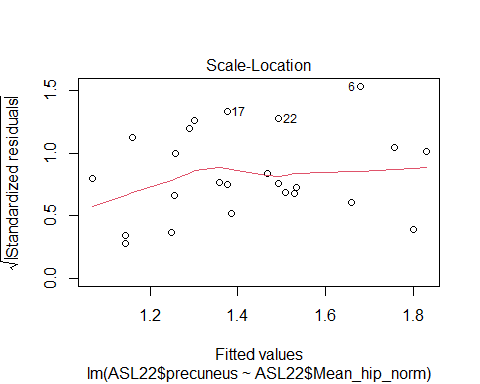

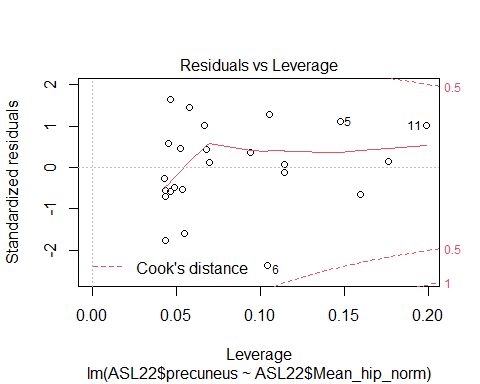


library(ggplot2)
ggplot(ASL22, aes(x=precuneus, y=Mean_hip_norm)) +
 geom_point() +
 geom_smooth(method=lm , color="red", fill="#69b3a2", se=TRUE) +
 xlab("Normalized rCBF in precuneus") + ylab("Normalized rCBF in hippocampus")

## `geom_smooth()` using formula = 'y ~ x'


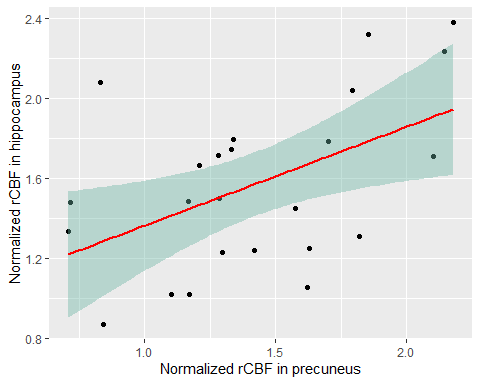

Supplement: Supplementary file 1 — Supplementary Material 1 [file 13195_2024_1432_MOESM1_ESM.docx]
